# Emerging agents that target signaling pathways in cancer stem cells

**DOI:** 10.1186/s13045-020-00901-6

**Published:** 2020-05-26

**Authors:** Yue Yang, Xiaoman Li, Ting Wang, Qianqian Guo, Tao Xi, Lufeng Zheng

**Affiliations:** 1 School of Life Science and Technology, Jiangsu Key Laboratory of Carcinogenesis and Intervention, China Pharmaceutical University, 639 Longmian Road, Nanjing, 211198 People’s Republic of China; 2 Jiangsu Key Laboratory for Pharmacology and Safety Evaluation of Chinese Materia Medica, School of Pharmacy, Nanjing University of Chinese Medicine, Nanjing, 210023 People’s Republic of China; 3 Department of Pharmacy, Affiliated Cancer Hospital of Zhengzhou University, Henan Cancer Hospital, 127 Dongming Road, Zhengzhou, 450003 People’s Republic of China

**Keywords:** Cancer stem cells, Small-molecule compounds, Wnt, Hedgehog, Notch, Hippo, Autophagy, Ferroptosis

## Abstract

Cancer stem cells (CSCs) contribute to the initiation, recurrence, and metastasis of cancer; however, there are still no drugs targeting CSCs in clinical application. There are several signaling pathways playing critical roles in CSC progression, such as the Wnt, Hedgehog, Notch, Hippo, and autophagy signaling pathways. Additionally, targeting the ferroptosis signaling pathway was recently shown to specifically kill CSCs. Therefore, targeting these pathways may suppress CSC progression. The structure of small-molecule drugs shows a good spatial dispersion, and its chemical properties determine its good druggability and pharmacokinetic properties. These characteristics make small-molecule drugs show a great advantage in drug development, which is increasingly popular in the market. Thus, in this review, we will summarize the current researches on the small-molecule compounds suppressing CSC progression, including inhibitors of Wnt, Notch, Hedgehog, and autophagy pathways, and activators of Hippo and ferroptosis pathways. These small-molecule compounds emphasize CSC importance in tumor progression and propose a new strategy to treat cancer in clinic via targeting CSCs.

## Background

**Fig. 1:**
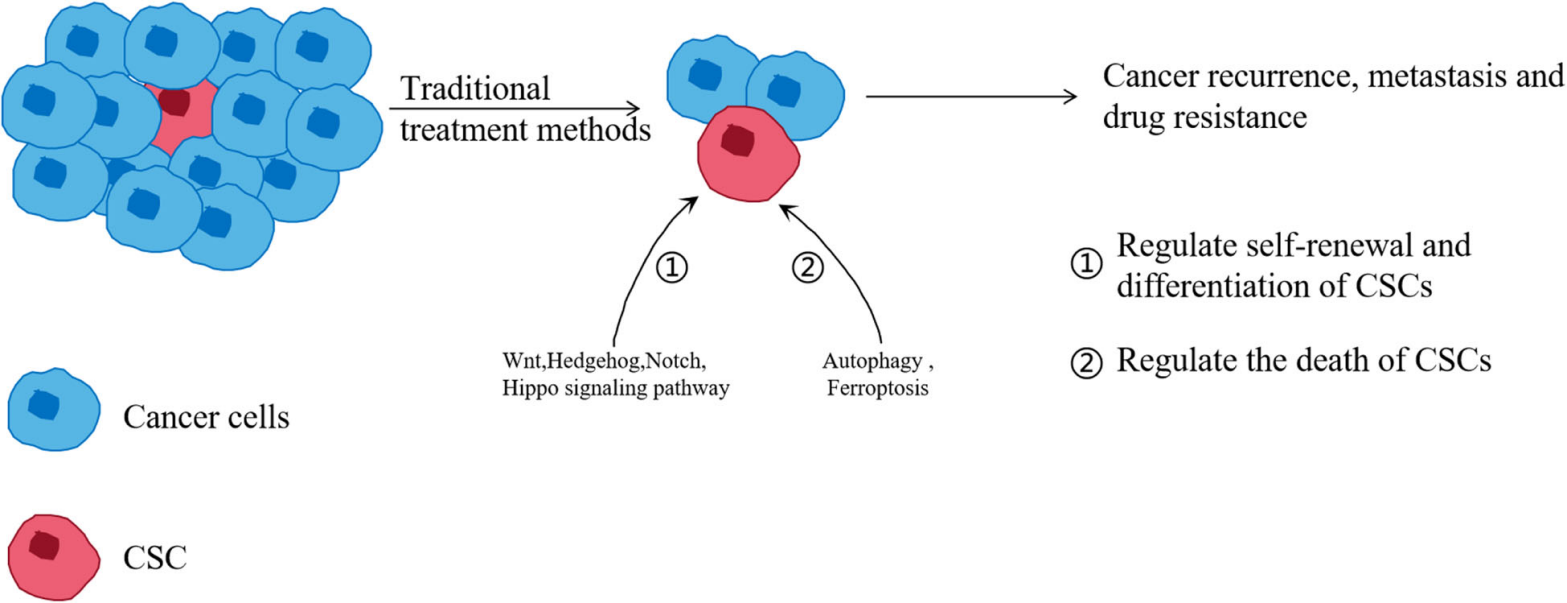
The mechanism whereby CSCs contribute to tumor recurrence, metastasis, and drug resistance. Traditional treatment methods (such as chemotherapy and radiotherapy) mainly target ordinary cancer cells but ignore the existence of CSCs. CSCs resist traditional therapy methods through various mechanisms, such as activating the signaling pathways involved in stemness and inhibiting cell death-related pathways

Although traditional therapeutic methods can reduce tumor volume extensively, cancer recurrence and metastasis always occur [1]. CSCs constitute a small portion of cancer cells, but they show resistance to chemotherapy and radiotherapy. Most conventional treatment methods kill cells with a proliferative potential to shrink the tumor but have no effect on stationary CSCs. This action explains why the tumor volume is reduced but the patient survival rate is not improved [2]. Due to their self-renewal ability and therapeutic resistance, CSCs are considered to be the root of tumor growth, recurrence, metastasis, and drug resistance [3]. Notably, because of their plasticity, stationary CSCs may produce cycling CSCs, which contributes to the relapse of cancer [4]. Therefore, more specific therapies targeting CSCs may lead to better results, and combining them with the traditional therapeutic methods may even achieve healing [5]. Cancer is a disease caused by disordered cell growth due to genetic mutations. CSCs have the same genetic driver mutations as most cancer cells, but CSCs have developmental characteristics that differ from those of non-stem cells, including differences in epigenetic modifications and gene expression profiles [6]. Many changes in the signaling pathways in CSCs provide a preliminary basis for developing compounds targeting CSCs. Signaling pathways that regulate the self-renewal and differentiation of CSCs include Wnt, Hedgehog (Hh), Notch, and Hippo, and these signaling pathways have been extensively studied [7, 8]. In addition, the signaling pathways inhibiting the proliferation of CSCs, in newly discovered mechanisms, have also gradually gained attention, and many promising results have been obtained (Fig. 1) [9]. Targeting CSCs with traditional therapy (chemotherapy and radiotherapy) may yield better results in controlling tumor growth, preventing cancer recurrence and metastasis, and decreasing drug resistance. Small-molecule compounds targeting CSCs play important role in this attenuation process. This review summarizes the research status of small-molecule compounds that suppress CSC progression, including inhibitors of the Wnt, Notch, Hh, and autophagy signaling pathways and activators of Hippo and ferroptosis signaling pathways, which may provide a basis for a current treatment for cancer.

## Signaling pathway regulators

CSCs display many characteristics of embryonic or tissue stem cells and often show continuous activation of one or more highly conserved signaling pathways related to development and tissue homeostasis. In these signaling pathways, the Wnt, Hh, Notch, and Hippo signaling pathways are associated with CSC self-renewal [10] and have been used to explore new drugs targeting CSCs.

### Wnt signaling pathway inhibitors

The Wnt signaling pathway is highly conserved among species and is divided into the β-catenin-dependent pathway and noncanonical Wnt pathway. When Wnt ligands bind to the Frizzled protein and low-density lipoprotein receptor-related protein (LRP) coreceptors, they initiate the transduction of Wnt/β-catenin signaling, eventually leading to β-catenin stabilization, nuclear translocation, and activation of target genes [31]. Since the β-catenin-dependent pathway has been extensively characterized in mammals, regulates the pluripotency of stem cells, and plays a critical role in self-renewal and differentiation ability, it is thought that abnormal activation of the Wnt pathway promotes CSC progression and thus leads to the deterioration and metastasis of cancer. Thus, the inhibition of CSCs can be mediated through this pathway [32, 33] (Table 1).

**Table 1:** Small-molecule compounds inhibiting CSC progression through suppressing Wnt signaling pathway

Name	Target	Mechanism	Type of cancer	Phase	NCT number (starting time)/publication date	Assessment
Wnt974	Wnt	Inhibits the proliferation of breast CSCs	Breast cancer	Phase I	NCT01351103(May 10, 2011)	Dysgeusia [11]
Niclosamide	Wnt/β-catenin	Selectively targets ovarian CSCs	Ovarian cancer	Preclinical	July, 2014	Without significant toxicity [12–14]
	LRP6, β-catenin	Decreases ALDH+ population cells	Basal-like breast cancer	Preclinical	April, 2014	
	Wnt/β-catenin	Suppresses CSC populations and self-renewal ability	Colorectal cancer	Phase II	NCT02519582 (August 11, 2015)	
ONC201	Wnt/β-catenin	Inhibits CSC self-renewal and deregulates CSC markers and CSC-related gene expression	Glioblastoma cancer	Phase I/II	NCT02038699 (January 16, 2014)	Well tolerated, Grade III neutropenia, Grade II allergic [15, 16]
			Prostate cancer	Preclinical	August 2, 2017	
XAV939	β-catenin	Attenuates CSC-mediated chemoresistance	Colon cancer	Preclinical	April, 2016	Induces cardiotoxicity and limited therapeutic window [17]
			HNSCC	Preclinical	October, 2019	
TFP	Wnt/β-catenin	Inhibits lung CSC spheroid formation and suppresses lung CSC marker expression (such as CD44/CD133)	Lung cancer	Preclinical	December 1, 2012	Induces little systemic toxicity, but grade 0–2 neurologic toxicity [18, 19]
Chelerythrine	β-catenin	Inhibits CSCs invasion, spheroid-forming ability, and the stem marker such as SOX2	NSCLC	Preclinical	January 6, 2020	Without systemic toxicity [20, 21]
FH535	Wnt/β-catenin	Deregulates pancreatic CSC marker CD24 and CD44 expression	Pancreatic cancer	Preclinical	July 26, 2016	Without side effects according to the current studies, still need experiments to prove [22]
Wnt-C59	Wnt	Decreases sphere formation of CSCs	NPC	Preclinical	June 10, 2015	Exhibits no apparent toxicity in mice; needs experiments to prove [23]
IWR-1	β-catenin	Impairs CSCs self-renewal and hampers the expression of key stem markers, and increases doxorubicin sensitivity	Osteosarcoma	Preclinical	February 1, 2018	Well tolerated in mice, but still needs to be thoroughly studied [24]
IC-2	Wnt	Reduces the population of CD44^+^ (liver CSCs) and the ability of sphere-forming ability	HCC	Preclinical	July, 2017	Not reported, still needs experiments to prove
	Wnt	Reduces the expression of CSC marker and sphere formation ability	CRC	Preclinical	August, 2017	
JIB-04	β-catenin	Inhibits the metastasis of colorectal CSCs	Colorectal cancer	Preclinical	April 26, 2018	Without general toxicity in JIB-04-treated mice, but still needs experiments to prove [25]
DTX and SFN	β-catenin	Inhibits the self-renewal ability of breast CSCs	Breast cancer	Preclinical	July, 2016	DTX has the side effect of neurological toxicity, nausea, diarrhea, and alopecia, but SFN is without significant toxicity [26, 27]
PP	β-catenin	Inhibits the self-renewal ability of breast CSCs	Breast cancer	Preclinical	March, 2016	PP shows no obvious toxicity in mice. But the poor targeting of it made the dosage large. It would be better to improve dosage form and develop new derivatives [28]
OXT-328					October, 2012	Safety according to the recent researches [28]
AD and Ts	Wnt/β-catenin	Decreases CSC number and activity, and reduces CSC marker expression (such as SOX2, ALDH1, and NOS2)	Lung cancer	Preclinical	December 3, 2019	AD has hepatoxicity, and TS is without toxic side effects in nude mice [29, 30]

In clinical trials, several small-molecule compounds have been used to target CSCs through the Wnt/β-catenin signaling pathway. For example, LGK-974 (Wnt974) can target porcupine to inhibit the post-translational acetylation of Wnt, thereby inhibiting Wnt secretion, and it has been reported that Wnt974 can inhibit the proliferation of breast CSCs (BCSCs) [34, 35]. Notably, in a phase I study, Wnt974 was found to be safe and effective in treating triple negative breast cancer (TNBC) (NCT01351103). Niclosamide has been approved by the FDA as an antihelminthic, and as a Wnt/β-catenin pathway inhibitor, it has anticancer ability that has been established by various studies. Niclosamide has been shown to selectively target ovarian CSCs [36]. In addition, niclosamide can decrease the population of ALDH^+^ cells by reducing the expression of LRP6 (a Wnt ligand receptor that plays a key role in the Wnt/β-catenin pathway) and β-catenin in basal-like breast cancer [37]. Notably, in a phase II trail, niclosamide was proved to safely and effectively treat colorectal cancer (CRC) [12]. Mechanistically, it can reduce the expression of many signaling components in the Wnt/β-catenin signaling pathway, the CSC population, and the self-renewal ability of CRC cells [38]. Additionally, ONC201, which is in a phase I/II study for patients with advanced cancer (NCT02038699), can inhibit CSC self-renewal and the expression of CSC-related genes in prostate and glioblastoma tumors through suppressing the Wnt signaling pathway [39].

Furthermore, there are many potential small-molecule compounds targeting CSCs through Wnt/β-catenin signaling pathway in preclinical experiments. For example, XAV939 can inhibit β-catenin signaling and thus attenuate CSC progression by interacting with the terminal anchor polymerase-binding domain (TBD) in Axin [40], which can abrogate CSC-mediated chemoresistance in head and neck squamous cell carcinoma (HNSCC) and colon cancer cells [41, 42]. Trifluoperazine (TFP) is used as an antipsychotic and an antiemetic, and it has been found to inhibit lung CSC spheroid formation ability and suppress lung CSC marker expression (such as CD44/CD133) by inhibiting Wnt/β-catenin signaling [43]. Chelerythrine chloride (Chelerythrine) can downregulate β-catenin and inhibit CSC invasion, spheroid-forming ability, and the expression of the stem marker SOX2 in non-small cell lung carcinoma (NSCLC) [44]. FH535 can suppress the expression of the pancreatic CSC marker CD24 and CD44 by inhibiting the Wnt/β-catenin signaling pathway [45]. Wnt-C59 (C59), an inhibitor of Wnt, can decrease the sphere formation ability of CSCs in a dose-dependent manner in nasopharyngeal carcinoma (NPC) [46]. IWR-1, a tankyrase inhibitor, can impair osteosarcoma CSC self-renewal, hamper the expression of key stem markers in osteosarcoma, and increase doxorubicin sensitivity in vivo by inhibiting β-catenin translocation [24]. IC-2, a novel small-molecule Wnt inhibitor, can reduce the population of CD44^+^ (liver CSCs) and the sphere-forming ability of hepatocellular carcinoma (HCC) cells [47]. It can also reduce the expression of CSC markers and the sphere formation ability in CRC. In addition, it can increase the sensitivity of 5-FU in the DLD-1 CRC cell line [48]. JIB-04, a selective inhibitor of histone demethylase, can inhibit the metastasis of colorectal CSCs by regulating the recruitment of β-catenin [49]. The combination of docetaxel (DTX) and sulforaphane (SFN), pyrvinium pamoate (PP), and phosphor-sulindac (OXT-328) can inhibit CSC self-renewal ability, the EMT (epithelial-mesenchymal transition), and drug resistance by decreasing β-catenin expression in BCSCs [50–52]. Additionally, actinomycin D (AD) and telmisartan (TS) can also decrease the CSC number and activity and reduce CSC marker expression (such as SOX2, ALDH1, and NOS2) in lung cancer by inhibiting the Wnt-β/catenin signaling pathway [53].

### Notch signaling pathway inhibitors

The Notch signaling pathway is an evolutionarily conserved pathway that is closely related to all aspects of cancer biology including CSC progression, angiogenesis, and tumor immunity [54]. The Notch pathway mainly consists of Notch receptors (Notch 1–4) and Notch ligands (Jagged 1, Jagged 2, delta-like ligand (DLL) -1, DLL-3, and DLL-4). When the receptors bind to the ligands, the Notch intracellular domain (NICD) is released into the nucleus through three cleavage processes mediated by γ-secretase, thereby activating the transcription of Notch target genes (Hes-1 and Hey-1) [55]. The activation of the Notch signaling pathway promotes tumor proliferation and metastasis; in contrast, inhibition of this pathway can eliminate CSCs and increase drug sensitivity. Therefore, genes in the Notch signaling pathway may represent potential cancer therapeutic targets [56]. Notch inhibitors can be used alone or in combination with chemotherapy agents to treat cancer and prevent recurrence [57].

Currently, inhibitors targeting the Notch signaling pathway mostly target γ-secretase or Notch ligands (Table 2). For example, MK-0752, a γ-secretase inhibitor, can decrease the population of CD44^+^/CD24^−^ and ALDH^+^, reduce mammosphere-forming efficiency, and inhibit tumor regeneration in BCSCs [63]. In a phase I study, the combined use of MK-0752 with docetaxel increased MK-0752-induced anti-BCSC ability and improved the efficiency of docetaxel in treating breast cancer (NCT00645333). PF-03084014, another γ-secretase inhibitor, can inhibit CSC self-renewal and proliferation and induce CSC differentiation by targeting Notch signaling pathways in HCC [64]. PF-03084014 can also decrease the CD44^+^/CD24^−^ and ALDH^+^ populations by suppressing N1ICD cleavage and the expression of Hes-1 and Hey-1 in pancreatic cancer [65]. In a phase II trail, PF-03084014 combined with gemcitabine and nab-paclitaxel increased the overall survival of patients with metastatic pancreatic adenocarcinoma (NCT02109445) compared to that of patients treated with PF-03084014. When combined with docetaxel, PF-03084014 can increase docetaxel efficiency against breast cancer; this combination is in a phase I study (NCT01876251). Mechanistically, PF-03084014 can diminish CD133^+^/CD44^+^ and ALDH^+^ subpopulations and eliminate BCSCs by targeting the Notch signaling pathway, thereby decreasing drug resistance [66].

**Table 2:** Small-molecule compounds inhibiting CSC progression through suppressing Notch signaling pathway

Name	Target	Mechanism	Type of cancer	Phase	NCT number (starting time)/publication date	Assessment
MK-0752	γ-secretase	Decreases the population of CD44^+^/CD24^−^ and ALDH^+^, reduces mammosphere-forming efficiency, and inhibits tumor regeneration in BCSCs	Breast cancer	Phase I	NCT00645333 (March 27, 2008)	Well tolerated, but exists dose-limiting toxicity (DLT) [58]
PF-03084014	γ-secretase	Inhibits CSC self-renewal and proliferation, and induces CSCs differentiation	HCC	Preclinical	August, 2017	Induces gastrointestinal toxicity and exists DLT [59]
	N1ICD, Hes-1, and Hey-1	Decreases CD44+/CD24− and ALDH+ population	Pancreatic cancer	Phase II	NCT02109445(April 9, 2014)	
	Notch	Diminishes CD133^+^/CD44^+^ and ALDH^+^ subpopulations and eliminates CSCs	Breast cancer	Phase I	NCT01876251(June 12, 2013)	
RO4929097	γ-secretase	Combined with 5-FU can decrease the proportion of CSC subgroup	INS	Preclinical	February, 2018	Fatigue is the most common toxicities, but it has DLT [60]
DAPT	Notch1	Inhibits the proliferation of LSCs and regulates LSC self-renewal	Leukemia	Preclinical	December, 2006	Induces low toxicity in cell and mice [61]
		Inhibits the self-renewal ability of ovarian CSCs and the expression of stem markers	Ovarian cancer	Preclinical	June, 2011	
Quinomycin A	Notch ligands	Inhibits pancreatic cancer microsphere formation, the stem marker and the number of CSCs	Pancreatic cancer	Preclinical	January 19, 2016	Induces gastrointestinal toxicity [62]

Additionally, the γ-secretase inhibitor RO4929097 can significantly inhibit Notch target genes Hes1 and Hey1, and it is in a phase II clinical setting to treat breast cancer, ovarian cancer, and renal cell carcinoma [67]. The combination of RO4929097 and 5-FU can decrease the proportion of the CSC subgroup with insulinomas (INS) [68]. DAPT (GSI-IX) is a new type of Notch1 inhibitor that was initially used to treat Alzheimer’s disease, and increasing number of studies have shown that it can inhibit CSCs [69]. Studies have demonstrated that DAPT can inhibit the proliferation and self-renewal ability of leukemia stem cells (LSCs) and ovarian CSCs [70, 71]. Additionally, quinomycin A can inhibit pancreatic cancer microsphere formation, stem marker expression, and CSC number by decreasing the expression of Notch ligands [72].

### Hh signaling pathway inhibitors

The Classical Hh signaling pathway is critical for embryonic development. The Hh signaling pathway regulates the self-renewal of CSCs and tissue homeostasis in cancer [80]. When extracellular Hh ligands (SHh, IH, and DHh) bind to PTCH, the inhibition of PTCH on Smoothened (SMO) is decreased, thereby GlI is translocated to the nucleus and induces the transcription of target genes [81]. Abnormal activation of the Hh signaling pathway is a crucial driver of breast cancer, prostate cancer, NSCLC, gastric cancer, and hematopoietic malignancies [82]. Hh signaling pathway inhibitors have been proven to be effective in early clinical trials. In addition, the development of Hh inhibitors has drawn significant interest for anticancer drug development. Recently, it has been reported that inhibition of the Hh signaling pathway can inhibit the self-renewal and drug resistance of pancreatic and breast CSCs [83, 84] (Table 3).

**Table 3:** Small-molecule compounds inhibiting CSC progression through suppressing Hh signalling pathway

Name	Target	Mechanism	Type of cancer	Phase	NCT number (starting time)/publication date	Assessment
Glasdegib	Hh	Attenuates the potential of leukemia-initiation and increases the sensitivity of LSCs to chemotherapy	Leukemia	Approved	November 21, 2018	Induces common side effect of chemotherapy drugs such as fatigue, nausea, and febrile neutropenia, but also has embryo-fetal toxicity [73]
Sonidegib	SMO	Downregulates the expression of CSC markers and increases the sensitivity to paclitaxel	Breast cancer	Phase I	NCT02027376 (January 6, 2014)	Induces myalgia, fatigue, and abnormal hepatic function, and gastrointestinal toxicity and alopecia are related to the dose of Sonidegib [74, 75]
Vismodegib	SMO	Inhibits BCSC self-renewal and mammosphere formation	Breast cancer	Phase II	NCT02694224 (February 29, 2016)	DLT, hyperbilirubinemia [76]
		Suppresses pancreatic CSC proliferation and survival	Pancreatic cancer	Phase II	NCT01064622(February 8, 2010)	
		Decreases the stem markers (such as CD44 and ALDH) of colon CSCs	Colorectal cancer	Phase II	NCT00636610(March 14, 2008)	
Ciclesonide	Hh	Inhibits the growth of lung CSCs	Lung cancer	Preclinical	February 4, 2020	Well tolerated, but as corticosteroid, it may inhibit bone growth [77]
Cyclopamine	SMO	Inhibits bladder CSC self-renewal	Bladder cancer	Preclinical	March 1, 2016	Induces holoprosencephaly, dystonia, and lethargy in rodents [78]
GANT61	GLI1 and GLI2	Decreases the CSC population	Breast cancer	Preclinical	May, 2017	No side effects in the mice according to the current studies [79]

For example, ciclesonide was approved by the FDA to treat asthma, and it was found that ciclesonide can inhibit the growth of lung CSCs through Hh signaling-mediated SOX2 regulation [85]. Sonidegib, an SMO antagonist, was approved by the FDA for the treatment of advanced basal cell carcinoma [86]. Recently, it was shown that sonidegib can downregulate the expression of CSC markers and increase the sensitivity of TNBC to paclitaxel, thus improving patient survival and reducing metastasis [87]. In addition, in a phase I study, sonidegib obtained a better result in advanced TNBC when it was combined with docetaxel (NCT02027376). Vismodegib (GDC-0449), another SMO inhibitor, was approved by the FDA to treat basal cell carcinoma [88]. Recently, many studies have found that vismodegib can inhibit BCSC self-renewal and mammosphere formation [89]. In a phase II trial, vismodegib was added to neoadjuvant chemotherapy for TNBC patients (NCT02694224). It can also suppress pancreatic CSC proliferation and survival by inhibiting Hh signaling pathways [90]. In a phase II trial, vismodegib combined with gemcitabine and nab-paclitaxel was used against untreated metastatic pancreatic cancer [91]. In another phase Ib/II trial, vismodegib plus gemcitabine was used to treat metastatic pancreatic cancer [92]. Additionally, vismodegib decreased the stem markers (such as CD44 and ALDH) of colon CSCs [93], and it was used to treat untreated metastatic CRC in a phase II trial [94]. These results suggest that vismodegib can target CSCs through the Hh signaling pathway. Furthermore, glasdegib (PF-04449913), an Hh signaling pathway inhibitor, was approved by the FDA to treat acute myeloid leukemia [73]. Glasdegib can attenuate the potential of leukemia-initiation and increase the sensitivity of LSCs to chemotherapy by inhibiting the Hh signaling pathway [95]. Cyclopamine is a natural compound that can specifically target SMO and inhibit the Hh signaling pathway [96], and it was reported that cyclopamine can inhibit bladder CSC self-renewal [97]. GANT61, another Hh inhibitor, can decrease the CSC population by downregulating the expression of GLI1 and GLI2 in ER (estrogen receptor)-positive breast cancer [98].

### Hippo pathway activators

The Hippo signaling pathway plays an essential role in CSC self-renewal, the EMT, and drug resistance. Upon activation of the Hippo signaling pathway, MST1/2 phosphorylate and activate LATS1/2. Then, LATS1/2 inactivate YAP/TAZ, which was subsequently translocated into the cytoplasm, and thus inhibits the expression of TEAD (TEA domain family member)-mediated genes, thereby suppressing CSC progression [106]. In contrast, inhibition of the Hippo signaling pathway activates YAP/TAZ, conferring CSC-like characteristics to the cell and leading to tumorigenesis [107]. YAP/TAZ-TEAD acts as a tumor promoter in the Hippo signaling pathway, while the other members of the Hippo signaling pathway are mostly tumor suppressor genes. Thus, targeting YAP/TAZ may serve as a strategy to inhibit CSCs (Table 4).

**Table 4:** Small-molecule compounds inhibiting CSC progression through activating Hippo pathway

Name	Target	Mechanism	Type of cancer	Phase	NCT number (starting time)/publication date	Assessment
Verteporfin	YAP/TAZ	Reduces the expression of CSC markers and suppresses CSC proliferation	Gastric and esophageal cancer	Preclinical	August 1, 2014	Without visible toxicity in the mice [99]
			gastric cancer	Preclinical	Apr 15, 2020	
Evodiamine	LATS1/2	Inhibits the proliferation of colon CSCs	Colon cancer	Preclinical	December 10, 2019	Induces low toxicity and still needs much experiments to prove [100]
Fluvastatin	YAP	Reduces the expression of CD44 and the characteristics of malignant mesothelioma stem cells	Malignant mesothelioma	Preclinical	January 28, 2017	Without any genotoxic, and relatively safe in patients [101, 102]
Atorvastatin	TAZ	Decreases MDA-MB 231 cells stemness-related features (such as the decrease of CD44+/CD24- subpopulation of cells)	Breast cancer	Phase II	NCT02416427(April 15, 2015)	Muscle loss [103]
CA3	YAP/TEAD	Suppresses tumor microsphere, formation and reduces the proportion of ALDH1+ cells	Esophageal adenocarcinoma	Preclinical	February, 2018	Without apparent toxicity in mice according to the current studies [104]
CPZ	YAP	Inhibits tumor microsphere-formation and stem marker expression	Breast cancer	Preclinical	April 1, 2019	Induces fatal hepatic failure [105]

For instance, verteporfin is a photosensitizer approved by the FDA, and it has garnered increasing interest for its anticancer role in gastric and esophageal cancer. Verteporfin can inhibit the transcriptional activity of YAP/TAZ-TEAD, reduce the expression of CSC markers, and suppress CSC proliferation [108, 109]. Evodiamine (Evo), which is isolated from the Chinese herb *Evodia rutaecarpa Benham*, can activate MST1/2-mediated phosphorylation of LATS1/2, which leads to YAP/TAZ phosphorylation and prevents YAP/TAZ translocation from the cytoplasm into the nucleus, and it has been shown that Evo can inhibit the proliferation of colon CSCs [110, 111]. Additionally, tanshinone IIA and limonin, which are extracted from Chinese herbs, have been shown to attenuate the stemness of cervical carcinoma stem cells by inhibiting the cytoplasmic-nuclear translocation of YAP [112, 113]. Moreover, statins such as fluvastatin can reduce the expression of CD44 by accelerating YAP phosphorylation, thereby reducing the characteristics of malignant mesothelioma stem cells and drug resistance [114]. Atorvastatin, another stain, can target TAZ in breast cancer, and is in a phase II trial (NCT02416427). Notably, atorvastatin can decrease the stemness of MDA-MB 231 cells, as evident by the decrease in the CD44^+^/CD24^−^ subpopulation of cells by inducing LATS1 expression and downregulating the expression of YAP/TAZ [115].

Recently, a new type of YAP inhibitor, CA3, was screened from a chemical library and found to attenuate the transcriptional activity of YAP/TEAD, and CA3 shows an excellent ability to target CSCs and inhibit tumor growth, as evident by its role in suppressing tumor sphere formation and reducing the proportion of ALDH1^+^ cells [104]. In addition, the antipsychotic drug chlorpromazine (CPZ) can kill breast cancer and BCSCs, which were characterized by inhibited tumor microsphere-formation and stem marker expression through promoting YAP degradation [116].

## Selective inducers of signaling pathways that contribute to cell death

According to measurable biochemical characteristics and molecular mechanisms, signaling pathways contributing to cell death mainly include apoptosis, autophagy, necroptosis, and ferroptosis. Cancer cells also undergo multiple forms of cell death during tumor development, including apoptosis, autophagy, and necrosis. Recently, ferroptosis has been shown to play a critical role in the development of cancer and may be a beneficial anticancer treatment strategy, which has gradually gained attention. Because classic apoptosis-inducing drugs have a poor effect on CSCs, current research is more intent on inhibiting CSCs by inducing autophagy and ferroptosis, which can effectively inhibit cancer recurrence and metastasis

### Autophagy and CSCs

Autophagy is a self-digesting mechanism in which proteins, lipids, and damaged organelles (such as mitochondria) are sequestered into vesicles called autophagosomes for degradation and recycling. Under physiological conditions, autophagy is critical for maintaining cell homeostasis and controlling protein and organelle quality. High levels of autophagy often occur in CSCs. Autophagy can help CSCs maintain their diversity and overcome low nutrients and hypoxia in the tumor microenvironment; therefore, it promotes CSCs to metastasis, drug resistance, and immune surveillance evasion [122]. Recently, autophagy was associated with CSC progression in breast cancer, NSCLC, prostate cancer, leukemia, gastric cancer, and myeloma, and its dysfunction affects the self-renewal ability of CSCs. Therefore, autophagy can be used as a CSC target. Although promotion of autophagy in CSCs is specific, different types, periods, and microenvironments have been shown to inhibit CSC progression through autophagy. For example, in some acute myeloid leukemias, many autophagy-related genes are mutated or downregulated in patients [123]. Therefore, currently, the compounds targeting autophagy mostly inhibit the autophagy of CSCs to suppress CSC progression (Table 5).

**Table 5:** Small-molecule compounds targeting autophagy to inhibit CSCs

Name	Target	Mechanism	Type of cancer	Phase	NCT number (starting time)/publication date	Assessment
CQ	Autophagy	Targets CSCs by inhibiting autophagy	Breast cancer	Phase II	NCT02333890 (January 7, 2015)	Induces cardiotoxicity [117]
	Autophagy	Inhibits the stemness marker of CD133+ and decreases the CSC proportions	NSCLC	Preclinical	December, 2019	
HCQ	Autophagy	Eliminates LSCs	Leukemia	Phase II	NCT00771056 (October 10, 2008)	Induces retinal toxicity [118]
Pantoprazole	Autophagy	Inhibits autophagy	Prostate cancer	Phase II	NCT01748500(December 12, 2012)	DLT, grade 3 to 4 [119]
	EMT/β-catenin	Inhibits the chemoresistance of gastric cancer stem cells	Gastric cancer	Preclinical	December, 2016	
3-MA	Autophagy	Reduces the resistance of mesenchymal stem cells	Myeloma	Preclinical	September, 2017	No significant side effect [120]
Rott	Autophagy	Induces autophagy leading to breast CSC death	Breast cancer	Preclinical	December 23, 2013	Without toxicity in the mice [121]

For example, chloroquine (CQ) is widely used in clinical antimalarial drugs and can be used in combination with anticancer drugs to treat cancer. CQ can effectively target CSCs by inhibiting autophagy, which causes the destruction of mitochondrial structures and double-stranded DNA, and the combination of CQ and carboplatin may be a useful adjuvant drug for treating TNBC [124]. CQ can inhibit the expression of stemness markers such as CD133 and decrease the proportions of CSCs, subsequently enhancing the efficacy of cisplatin against NSCLC by inhibiting autophagy [125]. In a phase II study, CQ inhibited breast cancer (NCT02333890). In addition, hydroxychloroquine (HCQ), a derivative of CQ, shows a stronger anticancer ability by targeting autophagy [126]. Additionally, the combination of HCQ and imatinib (IM) can effectively eliminate LSCs in chronic myeloid leukemia, and it is in a phase II trial [127]. A high dose of pantoprazole, a proton pump inhibitor (PPI), can affect docetaxel resistance in metastatic castration-resistant prostate cancer (mCRPC) by inhibiting autophagy, and it is in a phase II trial [128]. Moreover, pantoprazole can inhibit the chemoresistance of gastric CSCs [129]. Therefore, pantoprazole may target CSCs by inhibiting autophagy.

Moreover, concanamycin A is a selective inhibitor of V-ATPase that can inhibit the degradation of autolysosomes but has no effect on the formation of lysosomes; therefore, lysosomal degradation inhibitors may not reduce the rate of autophagosome-mediating chelation in destroying mitochondria. In this situation, some mitochondrial-dependent drugs may reduce the efficiency of inhibitors on CSCs; therefore, targeting early autophagy such as with VPS34 or ULK1 inhibitors may lead to better results. In addition, 3-methyladenine (3-MA) can prevent the formation of autolysosomes and reduce the resistance of mesenchymal stem cells; therefore, it can provide new treatment strategies for patients with multiple myeloma resistance [130]. Rottlerin (Rott), an active molecule that is isolated from *Mallotus philippensis*, is used in the treatment of allergies and helminthiasis and can induce autophagy, leading to BCSC death [131]. However, it is noteworthy that, although autophagy-targeting therapy has fewer side effects on normal cells than do conventional therapies, it also has some unknown effects that remain to be explored.

### Ferroptosis and CSCs

**Fig. 2:**
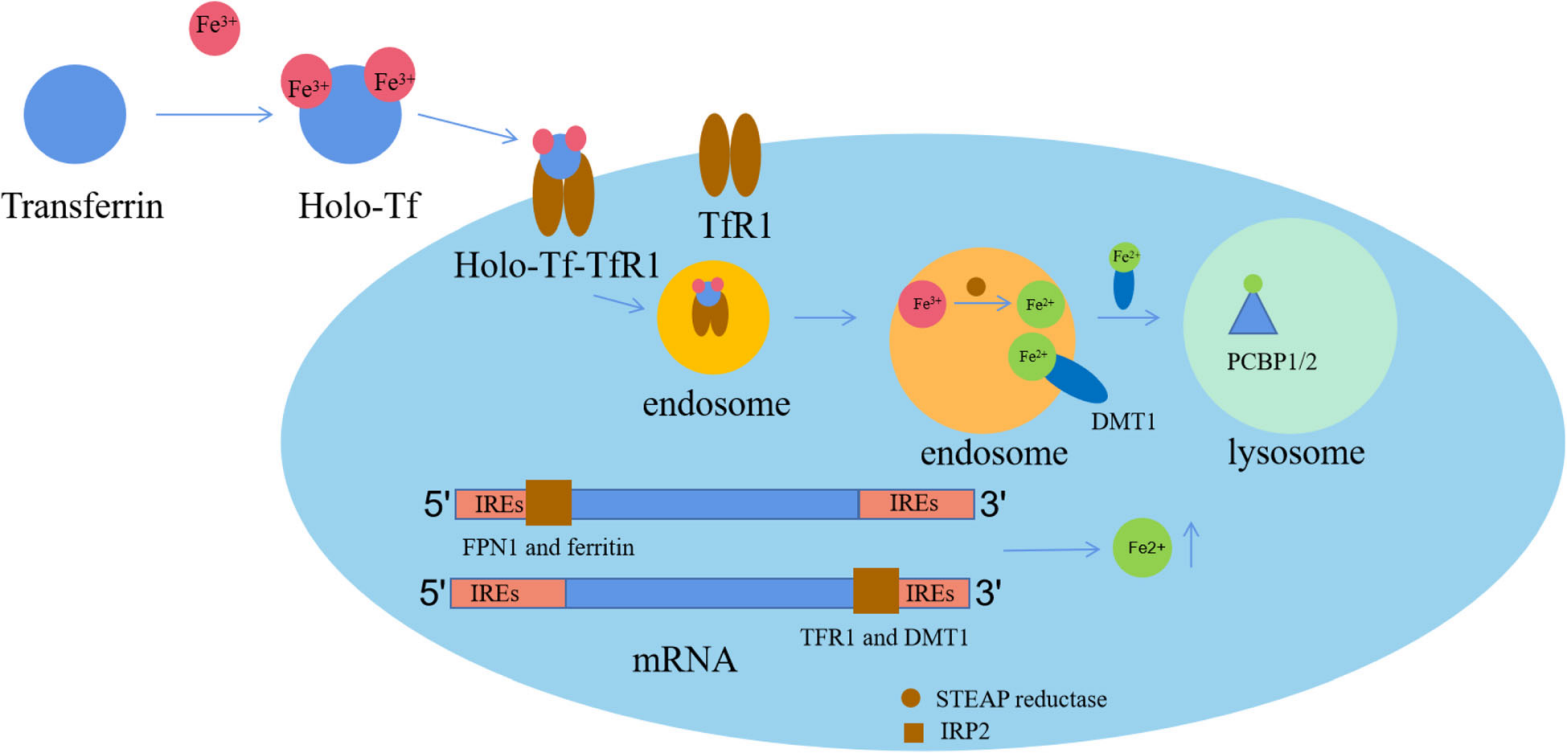
The regulation of iron. Transferrin can combine with two Fe^3+^ ions and then bind to transferrin receptor 1 (TfR1), forming the holo-Tf-TfR1 complex. This complex is encased in endosomes. In the endosome, Fe^3+^ is converted to Fe^2+^, which then binds to DMT1 (divalent metal transporter 1), forming the Fe^2+^-DMT1 complex. This complex is transferred to the labile iron pool (LIP)/lysosome, and Fe^2+^ binds to PCPB1/2 (poly-(rC)-binding protein 1/2). IRP2 binds to the 5'-end IREs in FPN1 and ferritin to inhibit their transcription and 3'-end IREs in TFR1 and DMT1 to prevent their degradation; therefore, the iron level in the cell increases

Ferroptosis is a new type of programmed cell death that is different from apoptosis, necrosis, and autophagy at the morphological and biochemical levels. It is induced by the accumulation of iron and lipid peroxidation caused by reactive oxygen species (ROS). The destruction of iron homeostasis and uncontrolled lipid peroxidation are two key features of ferroptosis [132]. The regulation of intracellular iron homeostasis is mainly regulated by IRP2 (iron regulatory protein 2). IRP2 can bind to iron-responsive elements (IREs) in mRNA 5'- and 3'-untranslated regions (UTRs) to inhibit transcription or stabilize mRNA. In the absence of iron, IRP2 binds to 5'-end IREs in FPN1 and ferritin to decrease their mRNA stability, and it binds to 3'-end IREs in TFR1 and DMT1 to stabilize mRNA. When there is excess iron in the cells, the synthesis of FPN1 and ferritin mRNA is inhibited and the degradation of TFR1 and DMT1 mRNA is enhanced (Fig. 2) [133].

**Fig. 3:**
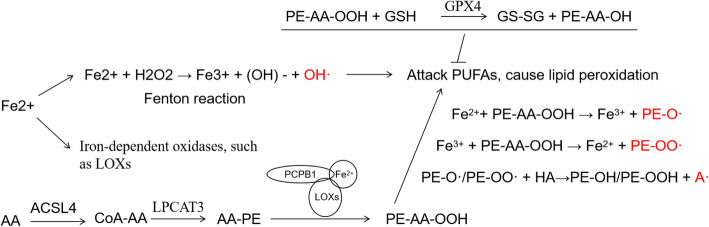
The role of iron in lipid peroxidation. Through the Fenton reaction, Fe^2+^ and hydrogen peroxide produces OH, and AA (arachidonic acid) can produce PE-AA-OOH through ACSL4 (acyl-CoA synthetase long-chain family member 4), LPCAT3 (lysophosphatidylcholine acyltransferase 3), and the complex of PCPB1, Fe^2+^, and LOXs (lipoxygenases). PE-AA-OOH, through Fe^2+^ + PE-AA-OOH → Fe^3+^ + PE-O, Fe^3+^ + PE-AA-OOH → Fe^2+^ + PE-OO, and PE-O/PE-OO + HA → PE-OH/PE-OOH + A produces PE-O, PE-OO, and A. OH, PE-O, PE-OO, and A can attack PUFAs (polyunsaturated fatty acids) to cause lipid peroxidation. In addition, GPX4 can inhibit the production of lipid peroxidation through PE-AA-OOH + GSH → GS-SG + PE-AA-OH

Additionally, the selenoenzyme glutathione peroxidase 4 (GPX4), which can inhibit the peroxidation of phospholipids (PLs), is considered to be a regulator of ferroptosis, and inactivation of GPX4 will lead to an increase in lipid peroxides. In addition, the accumulation of iron produces lipid peroxides in a nonenzymatic or enzyme-dependent manner [134]. The nonenzymatic reaction of iron to produce lipid peroxides is the Fenton reaction, Fe^2+^ + H_2_O_2_ → Fe^3+^ + (OH)^−^ + OH ·[135, 136] (Fig. 3). Another pathway for ferroptosis is mediated by the X_c_^−^ system, formed by SLC3A2 and SLC7A11, which internalizes cystine cells and expels glutamic acid, and then cystine is reduced to cysteine. Cysteine participates in the synthesis of GSH. GSH acts as an electron donor to maintain the activity of GPX4, thereby preventing ferroptosis.

#### Relationship between ferroptosis and CSCs

The changes in iron homeostasis in CSCs are usually manifested by high intracellular iron content. In addition, abnormal iron metabolism is associated with accelerated tumor growth and a poor prognosis for cancer patients. Therefore, because of the dependence of cancer on iron, iron-dependent mechanisms such as ferroptosis can be used as targets for drug development [137]. A higher level of iron in a CSC may affect its redox state, which is manifested by the increase in peroxidation and OH ·[138, 139].

Recently, it was reported that the levels of TfR1 and its ligand transferrin expressed by glioblastoma stem cells are higher than those expressed by non-CSCs, and iron-tracking experiments using these CSCs have shown that the level of iron intake by CSCs is greater than that internalized by non-CSCs [140], indicating that increased iron intake may also be a characteristic of CSCs. Additionally, ferritin is overexpressed in a variety of cancers, including breast cancer, pancreatic cancer, liver cancer, Hodgkin’s lymphoma, and glioblastoma. Ferritin may protect CSCs, but the degradation of ferritin produces a source of iron leading to ferroptosis. Targeting the H and L subunits of ferritin with siRNA causes a significant reduction in the growth of CSCs in vivo and in vitro [140].

#### Ferroptosis and small-molecule compounds

**Fig. 4:**
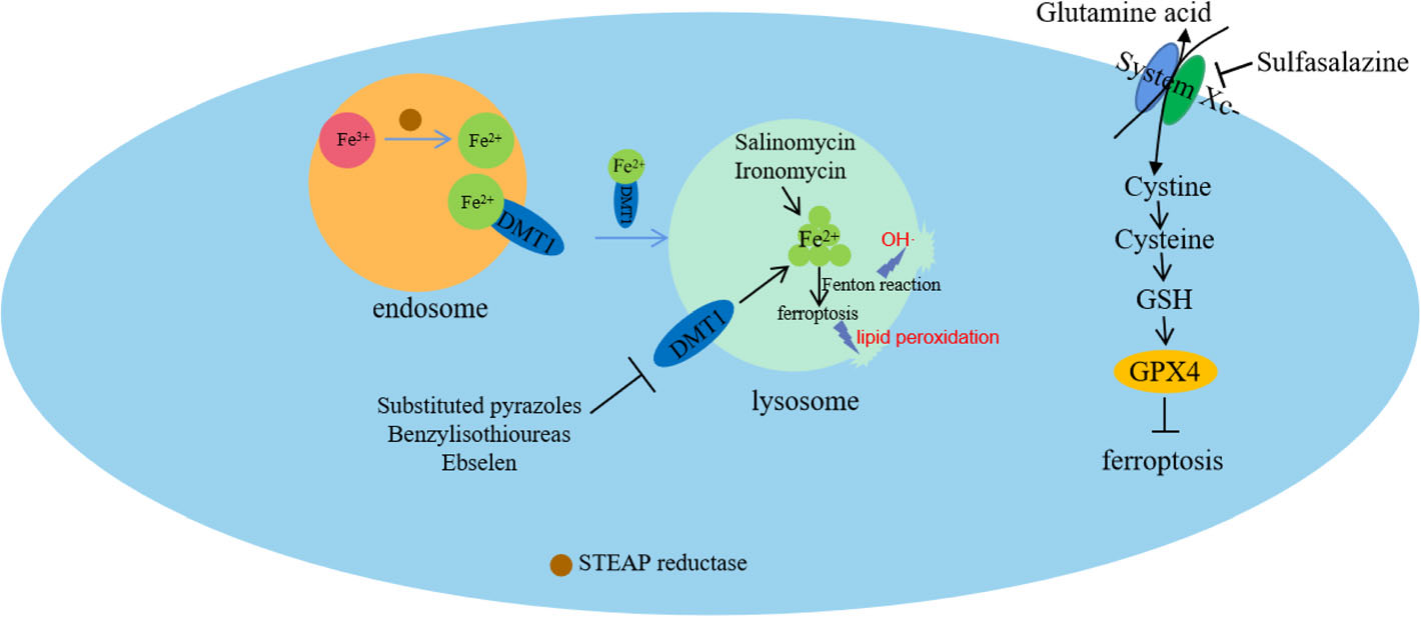
Summary of small-molecule compounds targeting ferroptosis to induce CSC death. Substituted pyrazoles, benzyl isothioureas, and ebselen can target DMT1 to block iron in lysosomes. Salinomycin and ironomycin can accumulate and isolate iron in the lysosome. Iron in lysosomes can produce OH through the Fenton reaction, and lipid peroxidation is also undertaken in the ferroptosis process. All the products can lead to increased permeability in the lysosomal membrane and cell death. Sulfasalazine can inhibit System Xc^−^. All these compounds can induce ferroptosis and thus kill CSCs

Current studies on the activators of ferroptosis are relatively extensive, but there are few small-molecule compounds that can target CSCs by mediating ferroptosis (Fig. 4). Additionally, the role of GPX4 in ferroptosis is decisive. Some experiments have already confirmed that the dysfunction of GPX4 causes mesenchymal stem cell ferroptosis; therefore, small-molecule compounds targeting GPX4 may induce ferroptosis in CSCs, although experimental proof is currently lacking.

The following is a summary of some ferroptosis inducers. Erastin, sulfasalazine, and sorafenib can reduce the level of cystine by inhibiting the X_c_^−^ system. Sulfasalazine can also inhibit the X_c_^−^ system, thereby inhibiting the progression of CSCs overexpression of CD44 in gastrointestinal cancer [141]. (1S, 3R)-RSL3, altretamine and withaferin A can increase lipid peroxidation by inhibiting or silencing GPX4 function [142]. Notably, salinomycin can increase the production of lipid peroxidation by blocking iron transport and depleting ferritin, by which it can specifically kill CSCs [143]. Other studies indicate that a combination of salinomycin and docetaxel can effectively kill gastric CSCs [144]. Salinomycin can effectively destroy non-CSCs and CSCs, as well as cancer cells with a multi-drug resistance (MDR) phenotype; therefore, it shows strong antitumor activity against a variety of cancers [145]. Additionally, salinomycin-loaded gold nanoparticles show enhanced ability to target BCSCs [146]. Considering the effect of salinomycin in killing CSCs, many salinomycin derivatives have also been developed and exhibit higher activity and selectivity, such as ironomycin and the products of C20-amination, C1-esterification, C9-oxidation, and C28-dehydration. These derivatives of salinomycin can accumulate and isolate iron in the lysosome, and the accumulation of iron in the lysosome initiates the Fenton reaction, leading to increased permeability in the lysosomal membrane and cell death. They have been shown to be at least ten-fold more potent than salinomycin in vivo and in vitro. In addition, ironomycin can effectively reduce the number of CSCs in docetaxel-resistant xenograft models [143, 147]. Furthermore, ebselen, substituting pyrazole, and benzyl isothiourea, which are the inhibitors of DMT1, can selectively target BCSCs by blocking iron in lysosomes [148]. These results indicate that increasing iron levels in lysosomes can initiate cell death in a manner similar to ferroptosis, and thus specifically and effectively kill CSCs. However, the concrete mechanisms by which the drugs target DMT1 are still unclear.

Notably, recent studies have shown that ovarian CSCs rely on iron for self-renewal and metastasis [149]. This finding reveals an opportunity to treat cancer by regulating iron balance. For example, after treatment with erastin, CSCs are more likely to induce ferroptosis than are non-CSCs [149]. The combination of temozolomide (TMZ) and CQ can cause glioblastoma stem cells (GSCs) to die in the form of ferroptosis, and specifically, this combination can reduce the self-renewal of GSCs, weaken the invasion of glioblastoma, and improve the therapeutic efficiency of chemotherapy and radiotherapy, but the specific target has not been identified [150].

Recently, an increasing number of studies have shown that artemisinin derivatives have anticancer ability. For example, dihydroartemisinin (DHA) can increase iron levels in the cell and inhibit the synthesis of ferritin through the IRP-IRE axis, which increases the concentration of intracellular iron, making cancer cells (such as lung, colorectal, and breast cancer cells) more sensitive to ferroptosis [151]. In addition, it was found that DHA can inhibit sphere formation and stem marker (CD133, SOX2, and nestin) expression in glioma CSCs [152]. Therefore, DHA may inhibit CSCs through ferroptosis. Through a platform of induced cancer stem-like cells (iCSCLs) used for high-throughput screening, artesunate can induce mitochondrial dysfunction in CSCs; therefore, it can inhibit the stemness of CSCs [153]. Artesunate can induce ferroptosis in pancreatic cancer [154]. All these findings indicate that artemisinin derivatives may target CSCs through ferroptosis. Ferumoxytol is a superparamagnetic iron oxide nanoparticle approved by the FDA, and its anticancer ability is associated with ferroptosis [155]. Magnetic hyperthermia is a new method by which to selectively kill CSCs (A549 and MDA-MB-231), and the key aspect of this technology is superparamagnetic iron oxide nanoparticles [156]. The report indicated that ferumoxytol was a new material for use in magnetic hyperthermia [157]. In addition, DOX@FMT-MC, a novel magnetic hydrogel complex consisting of ferumoxytol, doxorubicin, and chitosan, was applied in the clinic to treat colon carcinoma [158]. Therefore, ferumoxytol may target CSCs by ferroptosis (Table 6).

**Table 6:** Small-molecule compounds that induce ferroptosis to inhibit CSCs

Name	Target	Mechanism	Type of cancer	Phase	NCT number (starting time)/publication date	Assessment
Salinomycin	Ferroptosis	Increases the production of lipid peroxidation by blocking iron transport and depleting ferritin; these can specifically kill CSCs	Breast cancer	Preclinical	October, 2017	Induces neural and muscular toxicity; changing dosages and making chemical modifications may reduce toxicity [159]
	Ferroptosis	Combined with docetaxel can kill gastric CSCs	Gastric cancer	Preclinical	October, 2017	
Ironomycin	Ferroptosis	Reduces the number of CSCs in docetaxel-resistant xenograft models	Breast cancer	Preclinical	February 21, 2020	The potency against CSCs is ten-fold that of salinomycin; may cause nephrotoxicity and hepatotoxicity [160]
Ebselen	Ferroptosis	Targets BCSCs by blocking iron in lysosomes	Breast cancer	Preclinical	February 21, 2020	Induces low toxicity and shows good blood-brain barrier permeability and oral absorption [161]
Substituted pyrazoles					February 21, 2020	Not reported; need studies to prove
Benzylisothioureas					February 21, 2020	Hemoglobinopathy including thalassemia [162]
TMZ and CQ	Ferroptosis	Causes glioblastoma stem cells (GSCs) to die through a form of ferroptosis and reduce the self-renewal ability of GSCs	Glioblastoma	Preclinical	August 6, 2018	TMZ is well tolerated, but may induce hematological toxicity and infection; CQ shows cardiotoxicity [117, 163]
DHA	Ferroptosis	Ferroptosis	Lung, colorectal, and breast cancer cells	Preclinical	January, 2020	Induces neurotoxicity, cardiotoxicity and the toxicity in embryos [164]
	Apoptosis	Inhibits sphere formation and stem marker (CD133, SOX2, and nestin) expression in glioma CSCs	Gliomas	Preclinical	October, 2014	
Artesunate	Mitochondrial	Inhibits the stemness of CSCs	Not mentioned	Preclinical	September 2, 2016	Excellently tolerated, and with low adverse effects [165, 166]
	Ferroptosis	Induces cell death through ferroptosis	Pancreatic cancer	Preclinical	May 2, 2015	
Ferumoxytol	Ferroptosis	Selectively kills CSCs (A549 and MDA-MB-231 cells)	Lung cancer and breast cancer	Preclinical	August 26, 2013	Well tolerated, but intravenous may cause hypersensitivity, hypotension, and gastrointestinal side effects [167–169]
Sulfasalazine	System X_c_^−^	Inhibits the progression of CSCs overexpressing CD44	Gastrointestinal cancer	Preclinical	March 8, 2011	Induces gastrointestinal toxicity, and combed with other drugs, this side effect may be overcome [170]

According to the current research, the relationship between ferroptosis and CSCs has received increasing attention. In contrast, there are few reports of GPX4 in CSCs. Therefore, to some extent, the role of iron in CSCs is seemingly more dependent on GPX4 dysfunction, which causes normal stem cells to die in an iron-dependent manner [171, 172].

## Multiple-signaling pathway inhibitors targeting CSCs

As we stated above, there are many signaling pathways regulating CSC progression. Hence, the compounds that simultaneously target multiple signaling pathways critical for CSC progression may yield better results than single-target treatments in controlling tumor occurrence, recurrence, and drug resistance (Table 7). For example, Z-ajoene, a compound extracted from garlic, was confirmed to inhibit CSC sphere-forming ability in glioblastoma multiforme (GBM), and Notch-, Wnt-, and Hh-related genes ware changed after treatment with Z-ajoene. In addition, Z-ajoene induced no cytotoxicity in normal cells [173]. Poziotinib, a pan-human epidermal growth factor receptor (HER) inhibitor, can decrease ovarian CSC sphere formation ability by disrupting the Wnt, Notch, and Hh signaling pathways in epithelial ovarian cancer (EOC) [176]. According to clinical studies, patients may experience diarrhea and rash when treated with poziotinib [174]. However, considering the multiple targets and anti-CSC activity of poziotinib, more studies are needed in the future. Additionally, 6-shogaol reduces the number of CD44^+^/CD24^−^ cell subpopulation in BCSCs and inhibit their sphere-forming ability by inducing autophagy and inhibiting the Notch signaling pathway [177]. Importantly, 6-shogaol shows low toxicity induction in normal cells [175].

**Table 7:** Small-molecule compounds regulating multi-signaling pathways to inhibit CSCs

Name	Target	Mechanism	Type of cancer	Phase	NCT number (starting time)/publication date	Assessment
Z-ajoene	Notch, Wnt, and Hh	Inhibits CSC sphere-forming ability	Glioblastoma multiforme	Preclinical	July, 2014	Without cytotoxic in normal cells [173]
Poziotinib	Wnt, Notch, and Hh	Decreases ovarian CSC sphere formation ability	Epithelial ovarian cancer	Preclinical	May 21, 2020	Diarrhea and rash [174]
6-Shogaol	Notch and autophagy	Inhibits the number of CD44+/CD24− cell subpopulation and decreases sphere-forming ability	Breast cancer	Preclinical	September 10, 2015	Induces low toxicity in normal cells [175]

## Conclusions

**Fig. 5:**
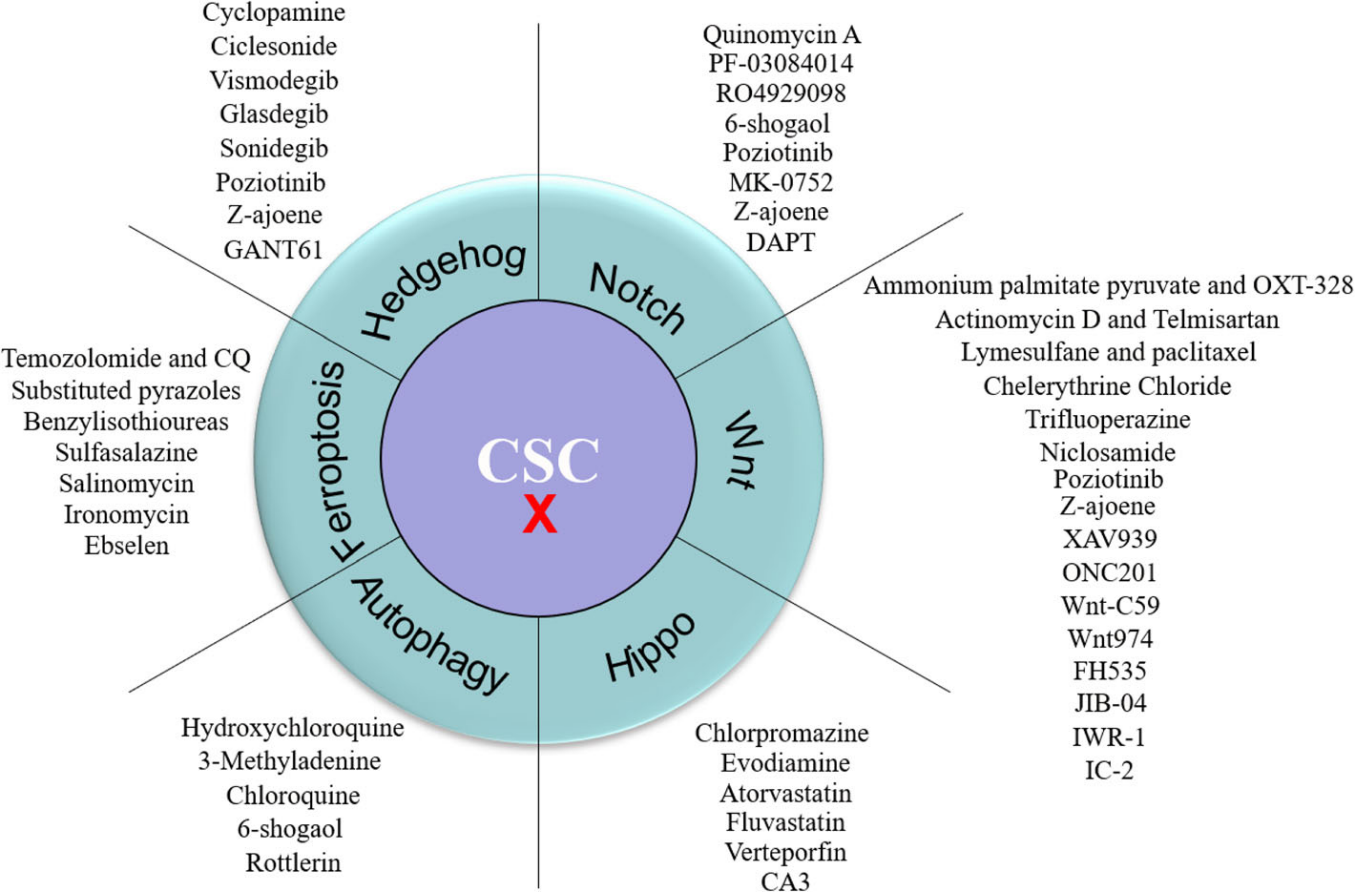
Summary of small-molecule compounds targeting CSCs. This figure lists all small-molecule compounds in this review targeting CSCs through different pathways

This review summarizes several well-characterized signaling pathways involved in CSCs, such as Wnt, Hh, Notch, Hippo, autophagy, and ferroptosis, and focuses on small-molecule compounds regulating these pathways (Fig. 5). Finally, these small-molecule compounds have the potential to kill CSCs, which provides a basis for cancer treatment. It is noteworthy that there are many other pathways, such as PI3K/Akt, MAPK, JAK/Stat, and TGF-β [178], essential for CSC survival, but these pathways are also widely engaged in other biological processes, and targeting them would not be specific to CSCs; however, the Wnt, Hh, Notch, and Hippo pathways are the mainstream pathways in CSCs, which have already been well studied. Therefore, in this current review, we summarize the small-molecule compounds targeting the Wnt, Hh, Notch, and Hippo pathways to kill CSCs. Although targeting ferroptosis to kill CSCs has recently been invoked, the effects have been strongly established by many studies [143, 147, 148]. Recently, although biologics have been rapidly developed, small-molecule compounds are still needed for clinical application.

Compared with biologics (such as monoclonal antibodies and antibody-drug conjugates), small-molecule compounds are inexpensive, and the generic drugs made from them are relatively simple, which means that small-molecule compounds may have the potential to compete with biologics. In addition, we present an overall assessment of these small-molecule compounds in Tables 1, 2, 3, 4, 5, and 6. Most chemotherapy drugs induce toxicity to different degrees. Therefore, we need to find a balance between anticancer effects and side effects of compounds used to target CSCs. The process from drug development to clinical use is long. Although approved drugs always induce specific toxicities, they have been well studied, and drug repurposing may offer a shortcut to prevent financial loss and detoured efforts. Therefore, in this review, we list many drugs approved by FDA, such as Wnt inhibitors (niclosamide, TFP, DTX and SFN, PP, AD and Ts); Notch inhibitors (DAPT); Hh inhibitor (glasdegib, sonidegib, vismodegib, ciclesonide); Hippo inhibitors (verteporfin, fluvastatin, atorvastatin, CPZ); autophagy regulators (CQ, HCQ, pantoprazole); ferroptosis inducers (TMZ and CQ, artesunate, ferumoxytol, sulfasalazine). These drugs display anti-CSC abilities, and some have been entered into clinical studies. In particular, an Hh inhibitor (glasdegib) has been approved by the FDA for its efficient anti-CSC action, which is similar to that of the biologics ELZONRIS. In addition, there are many new small-molecule compounds that are well tolerated, according to the current studies, but conclusive outcomes still need to be proven by more studies, and the toxicities of the following compounds have not been reported, which may be the result of insufficient experiments: Wnt inhibitors (ONC201, TFP, chelerythrine, FH535, Wnt-C59, IWR-1, IC-2, JIB-04, PP, OXT-328, OXT-328), Notch inhibitors (MK-0752, DAPT), Hh inhibitors (ciclesonide, GANT61), Hippo inhibitors (verteporfin, evodiamine, fluvastatin, CA3), autophagy regulators (3-MA, Rott), ferroptosis inducers (ebselen, substituted pyrazoles, TMZ, CQ, Artesunate, Ferumoxytol). Furthermore, we also listed some new CSC-targeting compounds that are in the preclinical or phase I/II stage: Wnt inhibitors (Wnt974, XAV939), Notch inhibitors (PF-03084014, RO4929097, quinomycin A), Hh inhibitor (cyclopamine), ferroptosis inducers (salinomycin, ironomycin, benzylisothioureas). These compounds also show anti-CSC activities, and the toxicities they induce may be reduced by changing the dosage, and developing new derivatives and combination therapies. To our surprise, some compounds are DLTs, especially the Notch inhibitors, and because of their side effect, additional research is needed to determine precise effective and safe dosage. In summary, according to the current studies, the compounds targeting CSCs are well tolerated in the mice or patients, and the compounds targeting multi-oncogenic signaling pathways may play important role in the clinic studies.

There are more detailed studies supporting the use of small-molecule compounds targeting Wnt, Hh, Notch, Hippo, and autophagy to inhibit CSCs, and many compounds are currently in clinical research. Although many small-molecule compounds activate ferroptosis, these studies have been conducted at the cell and mouse levels, and no clinical studies have been reported. However, based on the specificity and strong inhibitory ability of salinomycin and ironomycin and their derivatives on CSCs, these small-molecule compounds may have strong potential as target CSCs, and ferroptosis induced by iron in lysosomes may be a valuable prospect as a clinical target in CSCs to promote the development of anticancer drugs. However, it is noteworthy that there are different pathways in CSCs abnormally expressed; therefore, monotherapies that can target different pathways simultaneously or combined therapies may yield the best results in the future. Importantly, to our knowledge, targeting CSCs alone is sufficient for cancer therapy in the early stage of tumorigenesis, but it is not sufficient in the tumor development period, as many small-molecule compounds targeting CSCs cannot kill cancer cells, and the combined use of drugs targeting CSCs and chemotherapeutics may lead to better effects during tumor development period.

## Data Availability

Not applicable

## Abbreviations

CSCs: Cancer stem cells
Hh: Hedgehog
LRP: Lipoprotein receptor-related protein
TNBC: Triple negative breast cancer
TBD: Terminal anchor polymerase binding domain
HNSCC: Neck squamous cell carcinoma
CRC: Colorectal cancer
TFP: Trifluoperazine
NSCLC: Non-small cell lung carcinoma
NPC: Nasopharyngeal carcinoma
HCC: Hepatocellular carcinoma
DTX: Docetaxel
SFN: Sulforaphane
PP: Pyrvinium pamoate
EMT: Epithelial-mesenchymal transition
AD: Actinomycin D
TS: Telmisartan
DLL: Delta-like ligand
NICD: Notch intracellular domain
DLT: Dose-limiting toxicity
INS: Insulinomas
LSCs: Leukemia stem cells
SMO: Smoothened
ER: Estrogen receptor
BCSC: Breast cancer stem cell
TEAD: TEA domain family members
Evo: Evodiamine
CPZ: Chlorpromazine
CQ: Chloroquine
HCQ: Hydroxychloroquine
IM: Imatinib
mCRPC: Metastatic castration-resistant prostate cancer
PPI: Proton pump inhibitor
3-MA: 3-Methyladenine
Rott: Rottlerin
ROS: Reactive oxygen species
TfR1: Transferrin receptor 1
DMT1: Divalent metal transporter 1
LIP: Labile iron pool
PCBP1/2: Poly (rC)-binding protein 1/2
IRP2: Iron regulatory proteins 2
IREs: Iron-responsive elements
UTRs: Untranslated regions
Gpx4: Glutathione peroxidase 4
PLs: Phospholipids
PUFAs: Polyunsaturated fatty acids
LOXs: Lipoxygenases
AA: Arachidonic acid
ACSL4: Acyl-CoA synthetase long-chain family member
LPCAT3: Lysophosphatidylcholine acyltransferase 3
MDR: Multi-drug resistance
TMZ: Temozolomide
GSCs: Glioblastoma stem cells
DHA: Dihydroartemisinin
iCSCL: Induced cancer stem-like cells
GBM: Glioblastoma multiforme
HER: Pan-human epidermal growth factor receptor
EOC: Epithelial ovarian cancer

## References

[1] Chang JC (2016). Cancer stem cells: role in tumor growth, recurrence, metastasis, and treatment resistance. Medicine.

[2] Zhu Q, Shen Y, Chen X, He J, Liu J, Zu X (2020). Self-renewal signalling pathway inhibitors: perspectives on therapeutic approaches for cancer stem cells. OncoTargets and therapy.

[3] Nassar D, Blanpain C (2016). Cancer stem cells: basic concepts and therapeutic implications. Annu Rev Pathol.

[4] Katoh M (2017). Canonical and non-canonical WNT signaling in cancer stem cells and their niches: cellular heterogeneity, omics reprogramming, targeted therapy and tumor plasticity (Review). Int J Oncol.

[5] Reya T, Morrison SJ, Clarke MF, Weissman IL (2001). Stem cells, cancer, and cancer stem cells. Nature.

[6] Kreso A, Dick JE (2014). Evolution of the cancer stem cell model. Cell Stem Cell.

[7] An SM, Ding Q, Zhang J, Xie J, Li L (2014). Targeting stem cell signaling pathways for drug discovery: advances in the Notch and Wnt pathways. Sci China Life Sci.

[8] Takebe N, Miele L, Harris PJ, Jeong W, Bando H, Kahn M, Yang SX, Ivy SP (2015). Targeting Notch, Hedgehog, and Wnt pathways in cancer stem cells: clinical update. Nat Rev Clin Oncol.

[9] Bouvard C, Barefield C, Zhu S (2014). Cancer stem cells as a target population for drug discovery. Future Med Chem.

[10] Takebe N, Harris PJ, Warren RQ, Ivy SP (2011). Targeting cancer stem cells by inhibiting Wnt, Notch, and Hedgehog pathways. Nat Rev Clin Oncol.

[11] Solzak JP, Atale RV, Hancock BA, Sinn AL, Pollok KE, Jones DR, Radovich M (2017). Dual PI3K and Wnt pathway inhibition is a synergistic combination against triple negative breast cancer. NPJ breast cancer.

[12] Burock S, Daum S, Keilholz U, Neumann K, Walther W, Stein U (2018). Phase II trial to investigate the safety and efficacy of orally applied niclosamide in patients with metachronous or sychronous metastases of a colorectal cancer progressing after therapy: the NIKOLO trial. BMC Cancer.

[13] Lin CK, Bai MY, Hu TM, Wang YC, Chao TK, Weng SJ, Huang RL, Su PH, Lai HC (2016). Preclinical evaluation of a nanoformulated antihelminthic, niclosamide, in ovarian cancer. Oncotarget.

[14] Ye T, Xiong Y, Yan Y, Xia Y, Song X, Liu L, Li D, Wang N, Zhang L, Zhu Y (2014). The anthelmintic drug niclosamide induces apoptosis, impairs metastasis and reduces immunosuppressive cells in breast cancer model. PLoS One.

[15] Arrillaga-Romany I, Chi AS, Allen JE, Oster W, Wen PY, Batchelor TT (2017). A phase 2 study of the first imipridone ONC201, a selective DRD2 antagonist for oncology, administered every three weeks in recurrent glioblastoma. Oncotarget.

[16] Arrillaga-Romany I, Odia Y, Prabhu VV, Tarapore RS, Merdinger K, Stogniew M, Oster W, Allen JE, Mehta M, Batchelor TT (2020). Biological activity of weekly ONC201 in adult recurrent glioblastoma patients. Neuro-oncology.

[17] Liu M, Tu J, Gingold JA, Kong CSL, Lee DF (2018). Cancer in a dish: progress using stem cells as a platform for cancer research. Am J Cancer Res.

[18] Budd GT, Bukowski RM, Lichtin A, Bauer L, Van Kirk P, Ganapathi R (1993). Phase II trial of doxorubicin and trifluoperazine in metastatic breast cancer. Invest New Drugs.

[19] Li J, Yao QY, Xue JS, Wang LJ, Yuan Y, Tian XY, Su H, Wang SY, Chen WJ, Lu W (2017). Dopamine D2 receptor antagonist sulpiride enhances dexamethasone responses in the treatment of drug-resistant and metastatic breast cancer. Acta Pharmacol Sin.

[20] Chmura SJ, Dolan ME, Cha A, Mauceri HJ, Kufe DW, Weichselbaum RR (2000). In vitro and in vivo activity of protein kinase C inhibitor chelerythrine chloride induces tumor cell toxicity and growth delay in vivo. Clin Cancer Res.

[21] Medvetz D, Sun Y, Li C, Khabibullin D, Balan M, Parkhitko A, Priolo C, Asara JM, Pal S, Yu J (2015). High-throughput drug screen identifies chelerythrine as a selective inducer of death in a TSC2-null setting. Mol Cancer Res.

[22] Razak S, Afsar T, Almajwal A, Alam I, Jahan S (2019). Growth inhibition and apoptosis in colorectal cancer cells induced by vitamin D-nanoemulsion (NVD): involvement of Wnt/beta-catenin and other signal transduction pathways. Cell Biosci.

[23] Proffitt KD, Madan B, Ke Z, Pendharkar V, Ding L, Lee MA, Hannoush RN, Virshup DM (2013). Pharmacological inhibition of the Wnt acyltransferase PORCN prevents growth of WNT-driven mammary cancer. Cancer Res.

[24] Martins-Neves SR, Paiva-Oliveira DI, Fontes-Ribeiro C, Bovee J, Cleton-Jansen AM, Gomes CMF (2018). IWR-1, a tankyrase inhibitor, attenuates Wnt/beta-catenin signaling in cancer stem-like cells and inhibits in vivo the growth of a subcutaneous human osteosarcoma xenograft. Cancer Lett.

[25] Wang L, Chang J, Varghese D, Dellinger M, Kumar S, Best AM, Ruiz J, Bruick R, Pena-Llopis S, Xu J (2013). A small molecule modulates Jumonji histone demethylase activity and selectively inhibits cancer growth. Nat Commun.

[26] Kamal MM, Nazzal S (2018). Development of a new class of sulforaphane-enabled self-emulsifying drug delivery systems (SFN-SEDDS) by high throughput screening: a case study with curcumin. Int J Pharm.

[27] Kenmotsu H, Tanigawara Y (2015). Pharmacokinetics, dynamics and toxicity of docetaxel: why the Japanese dose differs from the Western dose. Cancer Sci.

[28] Xu W, Lacerda L, Debeb BG, Atkinson RL, Solley TN, Li L, Orton D, McMurray JS, Hang BI, Lee E (2013). The antihelmintic drug pyrvinium pamoate targets aggressive breast cancer. PLoS One.

[29] Koyama N, Nishida Y, Ishii T, Yoshida T, Furukawa Y, Narahara H (2014). Telmisartan induces growth inhibition, DNA double-strand breaks and apoptosis in human endometrial cancer cells. PLoS One.

[30] Lamture G, Crooks PA, Borrelli MJ (2018). Actinomycin-D and dimethylamino-parthenolide synergism in treating human pancreatic cancer cells. Drug Dev Res.

[31] de Sousa EMF, Vermeulen L. Wnt signaling in cancer stem cell biology. Cancers. 2016:8(7). 10.3390/cancers8070060 PMC4963802 27355964

[32] Curtin JC, Lorenzi MV (2018). Erratum: drug discovery approaches to target Wnt signaling in cancer stem cells. Oncotarget.

[33] Takahashi-Yanaga F, Kahn M (2010). Targeting Wnt signaling: can we safely eradicate cancer stem cells?. Clin cancer res.

[34] Takada R, Satomi Y, Kurata T, Ueno N, Norioka S, Kondoh H, Takao T, Takada S (2006). Monounsaturated fatty acid modification of Wnt protein: its role in Wnt secretion. Dev Cell.

[35] Zhao Z, Lu P, Zhang H, Xu H, Gao N (2014). Nestin positively regulates the Wnt/β-catenin pathway and the proliferation, survival and invasiveness of breast cancer stem cells. Breast Cancer Res.

[36] Arend RC, Londono-Joshi AI, Samant RS, Li Y, Conner M, Hidalgo B, Alvarez RD, Landen CN, Straughn JM, Buchsbaum DJ (2014). Inhibition of Wnt/beta-catenin pathway by niclosamide: a therapeutic target for ovarian cancer. Gynecol Oncol.

[37] Londono-Joshi AI, Arend RC, Aristizabal L, Lu W, Samant RS, Metge BJ, Hidalgo B, Grizzle WE, Conner M, Forero-Torres A (2014). Effect of niclosamide on basal-like breast cancers. Mol Cancer Ther.

[38] Park SY, Kim JY, Choi JH, Kim JH, Lee CJ, Singh P, Sarkar S, Baek JH, Nam JS (2019). Inhibition of LEF1-mediated DCLK1 by niclosamide attenuates colorectal cancer stemness. Clin Cancer Res.

[39] Prabhu VV, Lulla AR, Madhukar NS, Ralff MD, El-Deiry WS (2017). Cancer stem cell-related gene expression as a potential biomarker of response for first-in-class imipridone ONC201 in solid tumors. Plos One.

[40] Huang SMA, Mishina YM, Liu S, Cheung A, Stegmeier F, Michaud GA, Charlat O, Wiellette E, Zhang Y, Wiessner S (2009). Tankyrase inhibition stabilizes axin and antagonizes Wnt signalling. Nature.

[41] Roy S, Roy S, Kar M, Chakraborty A, Kumar A, Delogu F, Asthana S, Hande MP, Banerjee B (2019). Combined treatment with cisplatin and the tankyrase inhibitor XAV-939 increases cytotoxicity, abrogates cancer-stem-like cell phenotype and increases chemosensitivity of head-and-neck squamous-cell carcinoma cells. Mutat Res.

[42] Wu X, Luo F, Li J, Zhong X, Liu K (2016). Tankyrase 1 inhibitior XAV939 increases chemosensitivity in colon cancer cell lines via inhibition of the Wnt signaling pathway. Int J Oncol.

[43] Yeh CT, Wu AT, Chang PM, Chen KY, Yang CN, Yang SC, Ho CC, Chen CC, Kuo YL, Lee PY (2012). Trifluoperazine, an antipsychotic agent, inhibits cancer stem cell growth and overcomes drug resistance of lung cancer. Am J Respir Crit Care Med.

[44] Heng WS, Cheah SC (2020). Chelerythrine chloride downregulates beta-catenin and inhibits stem cell properties of non-small cell lung carcinoma. Molecules.

[45] Liu L, Zhi Q, Shen M, Gong FR, Zhou BP, Lian L, Shen B, Chen K, Duan W, Wu MY (2016). FH535, a beta-catenin pathway inhibitor, represses pancreatic cancer xenograft growth and angiogenesis. Oncotarget.

[46] Cheng Y, Phoon YP, Jin X, Chong SY, Ip JC, Wong BW, Lung ML (2015). Wnt-C59 arrests stemness and suppresses growth of nasopharyngeal carcinoma in mice by inhibiting the Wnt pathway in the tumor microenvironment. Oncotarget.

[47] Seto K, Sakabe T, Itaba N, Azumi J, Oka H, Morimoto M, Umekita Y, Shiota G (2017). A novel small-molecule WNT inhibitor, IC-2, has the potential to suppress liver cancer stem cells. Anticancer Res.

[48] Urushibara S, Tsubota T, Asai R, Azumi J, Ashida K, Fujiwara Y, Shiota G (2017). WNT/beta-catenin signaling inhibitor IC-2 suppresses sphere formation and sensitizes colorectal cancer cells to 5-fluorouracil. Anticancer Res.

[49] Kim MS, Cho HI, Yoon HJ, Ahn YH, Park EJ, Jin YH, Jang YK (2018). JIB-04, a small molecule histone demethylase inhibitor, selectively targets colorectal cancer stem cells by inhibiting the Wnt/beta-catenin signaling pathway. Sci Rep.

[50] Huang J, Tao C, Yu Y, Yu F, Zhang H, Gao J, Wang D, Chen Y, Gao J, Zhang G (2016). Simultaneous targeting of differentiated breast cancer cells and breast cancer stem cells by combination of docetaxel- and dulforaphane-loaded self-assembled poly(D, L-lactide-co-glycolide)/hyaluronic acid block copolymer-based nanoparticles. J Biomed Nanotechnol.

[51] Liang XU, Zhang LE, Chun HU, Liang S, Fei X (2016). WNT pathway inhibitor pyrvinium pamoate inhibits the self-renewal and metastasis of breast cancer stem cells. Int J Oncol.

[52] Zhu Caihua, Cheng Ka-Wing, Ouyang Nengtai, Huang Liqun, Sun Yu, Constantinides Panayiotis, Rigas Basil (2012). Phosphosulindac (OXT-328) Selectively Targets Breast Cancer Stem Cells In Vitro and in Human Breast Cancer Xenografts. STEM CELLS.

[53] Green R, Howell M, Khalil R, Nair R, Yan J, Foran E, Katiri S, Banerjee J, Singh M, Bharadwaj S (2019). Actinomycin D and telmisartan combination targets lung cancer stem cells through the Wnt/beta catenin pathway. Sci Rep.

[54] Espinoza I, Miele L (2013). Notch inhibitors for cancer treatment. Pharmacol Ther.

[55] Schweisguth F (2004). Regulation of notch signaling activity. Current biology : CB.

[56] Nefedova Y, Sullivan DM, Bolick SC, Dalton WS, Gabrilovich DI (2008). Inhibition of Notch signaling induces apoptosis of myeloma cells and enhances sensitivity to chemotherapy. Blood.

[57] Venkatesh Vandana, Nataraj Raghu, Thangaraj Gopenath S., Karthikeyan Murugesan, Gnanasekaran Ashok, Kaginelli Shanmukhappa B., Kuppanna Gobianand, Kallappa Chandrashekrappa Gowdru, Basalingappa Kanthesh M. (2018). Targeting Notch signalling pathway of cancer stem cells. Stem Cell Investigation.

[58] Krop I, Demuth T, Guthrie T, Wen PY, Mason WP, Chinnaiyan P, Butowski N, Groves MD, Kesari S, Freedman SJ (2012). Phase I pharmacologic and pharmacodynamic study of the gamma secretase (Notch) inhibitor MK-0752 in adult patients with advanced solid tumors. J Clin Oncol.

[59] Samon JB, Castillo-Martin M, Hadler M, Ambesi-Impiobato A, Paietta E, Racevskis J, Wiernik PH, Rowe JM, Jakubczak J, Randolph S (2012). Preclinical analysis of the gamma-secretase inhibitor PF-03084014 in combination with glucocorticoids in T-cell acute lymphoblastic leukemia. Mol Cancer Ther.

[60] Tolcher AW, Messersmith WA, Mikulski SM, Papadopoulos KP, Kwak EL, Gibbon DG, Patnaik A, Falchook GS, Dasari A, Shapiro GI (2012). Phase I study of RO4929097, a gamma secretase inhibitor of Notch signaling, in patients with refractory metastatic or locally advanced solid tumors. J Clin Oncol.

[61] Zhou Y, Gregor VE, Sun Z, Ayida BK, Winters GC, Murphy D, Simonsen KB, Vourloumis D, Fish S, Froelich JM (2005). Structure-guided discovery of novel aminoglycoside mimetics as antibacterial translation inhibitors. Antimicrob Agents Chemother.

[62] Harvey JH, McFadden M, Andrews WG, Byrne PJ, Ahlgren JD, Woolley PV (1985). Phase I study of echinomycin administered on an intermittent bolus schedule. Cancer Treat Rep.

[63] Schott AF, Landis MD, Dontu G, Griffith KA, Layman RM, Krop I, Paskett LA, Wong H, Dobrolecki LE, Lewis MT (2013). Preclinical and clinical studies of gamma secretase inhibitors with docetaxel on human breast tumors. Clin Cancer Res.

[64] Wu CX, Xu A, Zhang CC, Olson P, Chen L, Lee TK, Cheung TT, Lo CM, Wang XQ (2017). Notch inhibitor PF-03084014 inhibits hepatocellular carcinoma growth and metastasis via suppression of cancer stemness due to reduced activation of Notch1-Stat3. Mol Cancer Ther.

[65] Yabuuchi S, Pai SG, Campbell NR, de Wilde RF, De Oliveira E, Korangath P, Streppel MM, Rasheed ZA, Hidalgo M, Maitra A (2013). Notch signaling pathway targeted therapy suppresses tumor progression and metastatic spread in pancreatic cancer. Cancer Lett.

[66] Zhang CC, Yan Z, Zong Q, Fang DD, Painter C, Zhang Q, Chen E, Lira ME, John-Baptiste A, Christensen JG (2013). Synergistic effect of the gamma-secretase inhibitor PF-03084014 and docetaxel in breast cancer models. Stem Cells Transl Med.

[67] Chanh H, Laura P, SM F, Ratna M, Adele H, Silvia M, Shulian S, Anna P, Yongzhao S, Farbod D (2011). The novel gamma secretase inhibitor RO4929097 reduces the tumor initiating potential of melanoma. Plos One.

[68] Capodanno Y, Buishand FO, Pang LY, Kirpensteijn J, Mol JA, Argyle DJ (2018). Notch pathway inhibition targets chemoresistant insulinoma cancer stem cells. Endocr Relat Cancer.

[69] Zhao ZL, Zhang L, Huang CF, Ma SR, Bu LL, Liu JF, Yu GT, Liu B, Gutkind JS, Kulkarni AB (2016). NOTCH1 inhibition enhances the efficacy of conventional chemotherapeutic agents by targeting head neck cancer stem cell. Sci Rep.

[70] Gal H, Amariglio N, Trakhtenbrot L, Jacob-Hirsh J, Margalit O, Avigdor A, Nagler A, Tavor S, Ein-Dor L, Lapidot T (2006). Gene expression profiles of AML derived stem cells; similarity to hematopoietic stem cells. Leukemia.

[71] Jiang LY, Zhang XL, Du P, Zheng JH (2011). gamma-Secretase inhibitor, DAPT inhibits self-renewal and stemness maintenance of ovarian cancer stem-like cells in vitro. Chinese journal of cancer research = Chung-kuo yen cheng yen chiu.

[72] Ponnurangam S, Dandawate PR, Dhar A, Tawfik OW, Parab RR, Mishra PD, Ranadive P, Sharma R, Mahajan G, Umar S (2016). Quinomycin A targets Notch signaling pathway in pancreatic cancer stem cells. Oncotarget.

[73] Norsworthy KJ, By K, Subramaniam S, Zhuang L, Del Valle PL, Przepiorka D, Shen YL, Sheth CM, Liu C, Leong R (2019). FDA approval summary: glasdegib for newly diagnosed acute myeloid leukemia. Clin Cancer Res.

[74] Chen L, Silapunt S, Migden MR (2016). Sonidegib for the treatment of advanced basal cell carcinoma: a comprehensive review of sonidegib and the BOLT trial with 12-month update. Future Oncol.

[75] Minami H, Ando Y, Ma BB, Hsiang Lee J, Momota H, Fujiwara Y, Li L, Fukino K, Ito K, Tajima T (2016). Phase I, multicenter, open-label, dose-escalation study of sonidegib in Asian patients with advanced solid tumors. Cancer Sci.

[76] Abou-Alfa GK, Lewis LD, LoRusso P, Maitland M, Chandra P, Cheeti S, Colburn D, Williams S, Simmons B, Graham RA (2017). Pharmacokinetics and safety of vismodegib in patients with advanced solid malignancies and hepatic impairment. Cancer Chemother Pharmacol.

[77] Belvisi Maria G., Bundschuh Daniela S., Stoeck Michael, Wicks Sharon, Underwood Stephen, Battram Clifford H., Haddad El-Bdaoui, Webber Stephen E., Foster Martyn L. (2005). Preclinical Profile of Ciclesonide, a Novel Corticosteroid for the Treatment of Asthma. Journal of Pharmacology and Experimental Therapeutics.

[78] Everson JL, Sun MR, Fink DM, Heyne GW, Melberg CG, Nelson KF, Doroodchi P, Colopy LJ, Ulschmid CM, Martin AA (2019). Developmental toxicity assessment of piperonyl butoxide exposure targeting sonic hedgehog signaling and forebrain and face morphogenesis in the mouse: an in vitro and in vivo study. Environ Health Perspect.

[79] Lauth M, Bergstrom A, Shimokawa T, Toftgard R (2007). Inhibition of GLI-mediated transcription and tumor cell growth by small-molecule antagonists. Proc Natl Acad Sci U S A.

[80] Habib JG, O'Shaughnessy JA (2016). The hedgehog pathway in triple-negative breast cancer. Cancer Med.

[81] Merchant AA, Matsui W (2010). Targeting Hedgehog--a cancer stem cell pathway. Clin Cancer Res.

[82] Amakye D, Jagani Z, Dorsch M (2013). Unraveling the therapeutic potential of the Hedgehog pathway in cancer. Nat Med.

[83] Huang FT, Zhuan-Sun YX, Zhuang YY, Wei SL, Tang J, Chen WB, Zhang SN (2012). Inhibition of hedgehog signaling depresses self-renewal of pancreatic cancer stem cells and reverses chemoresistance. Int J Oncol.

[84] Liu S, Dontu G, Mantle ID, Patel S, Ahn NS, Jackson KW, Suri P, Wicha MS (2006). Hedgehog signaling and Bmi-1 regulate self-renewal of normal and malignant human mammary stem cells. Cancer Res.

[85] Choi HS, Kim SL, Kim JH, Lee DS (2020). The FDA-approved anti-asthma medicine ciclesonide inhibits lung cancer stem cells through Hedgehog signaling-mediated SOX2 regulation. Int J Mol Sci.

[86] Casey D, Demko S, Shord S, Zhao H, Chen H, He K, Putman A, Helms W, Keegan P, Pazdur R (2017). FDA approval summary: sonidegib for locally advanced basal cell carcinoma. Clin Cancer Res.

[87] Cazet AS, Hui MN, Elsworth BL, Wu SZ, Roden D, Chan CL, Skhinas JN, Collot R, Yang J, Harvey K (2018). Targeting stromal remodeling and cancer stem cell plasticity overcomes chemoresistance in triple negative breast cancer. Nat Commun.

[88] Ingram I (2012). Vismodegib granted FDA approval for treatment of basal cell carcinoma.

[89] Li W, Yang H, Li X, Han L, Xu N, Shi A (2019). Signaling pathway inhibitors target breast cancer stem cells in triple-negative breast cancer. Oncol Rep.

[90] Singh BN, Fu J, Srivastava RK, Shankar S (2011). Hedgehog signaling antagonist GDC-0449 (Vismodegib) inhibits pancreatic cancer stem cell characteristics: molecular mechanisms. PLoS One.

[91] De Jesus-Acosta A, Sugar EA, O'Dwyer PJ, Ramanathan RK, Von Hoff DD, Rasheed Z, Zheng L, Begum A, Anders R, Maitra A (2020). Phase 2 study of vismodegib, a hedgehog inhibitor, combined with gemcitabine and nab-paclitaxel in patients with untreated metastatic pancreatic adenocarcinoma. Br J Cancer.

[92] Catenacci DV, Junttila MR, Karrison T, Bahary N, Horiba MN, Nattam SR, Marsh R, Wallace J, Kozloff M, Rajdev L (2015). Randomized phase Ib/II study of gemcitabine plus placebo or vismodegib, a Hedgehog pathway inhibitor, in patients with metastatic pancreatic cancer. J Clin Oncol.

[93] Wu C, Hu S, Cheng J, Wang G, Tao K (2017). Smoothened antagonist GDC-0449 (Vismodegib) inhibits proliferation and triggers apoptosis in colon cancer cell lines. Exp Ther Med.

[94] Berlin J, Bendell JC, Hart LL, Firdaus I, Gore I, Hermann RC, Mulcahy MF, Zalupski MM, Mackey HM, Yauch RL (2013). A randomized phase II trial of vismodegib versus placebo with FOLFOX or FOLFIRI and bevacizumab in patients with previously untreated metastatic colorectal cancer. Clin Cancer Res.

[95] Fukushima N, Minami Y, Kakiuchi S, Kuwatsuka Y, Hayakawa F, Jamieson C, Kiyoi H, Naoe T (2016). Small-molecule Hedgehog inhibitor attenuates the leukemia-initiation potential of acute myeloid leukemia cells. Cancer Sci.

[96] Chen JK, Taipale J, Young KE, Maiti T, Beachy PA (2002). Small molecule modulation of Smoothened activity. Proc Natl Acad Sci U S A.

[97] Li C, Du Y, Yang Z, He L, Wang Y, Hao L, Ding M, Yan R, Wang J, Fan Z (2016). GALNT1-mediated glycosylation and activation of sonic Hedgehog signaling maintains the self-renewal and tumor-initiating capacity of bladder cancer stem cells. Cancer Res.

[98] Kurebayashi J, Koike Y, Ohta Y, Saitoh W, Yamashita T, Kanomata N, Moriya T (2017). Anti-cancer stem cell activity of a hedgehog inhibitor GANT61 in estrogen receptor-positive breast cancer cells. Cancer Sci.

[99] Michy T, Massias T, Bernard C, Vanwonterghem L, Henry M, Guidetti M, Royal G, Coll JL, Texier I, Josserand V (2019). Verteporfin-loaded lipid nanoparticles improve ovarian cancer photodynamic therapy in vitro and in vivo. Cancers.

[100] Su T, Yang X, Deng JH, Huang QJ, Huang SC, Zhang YM, Zheng HM, Wang Y, Lu LL, Liu ZQ (2018). Evodiamine, a novel NOTCH3 methylation stimulator, significantly suppresses lung carcinogenesis in vitro and in vivo. Front Pharmacol.

[101] Lopez-Aguilar E, Sepulveda-Vildosola AC, Rivera-Marquez H, Cerecedo-Diaz F, Valdez-Sanchez M, Villasis-Keever MA (1999). Security and maximal tolerated doses of fluvastatin in pediatric cancer patients. Arch Med Res.

[102] Robison RL, Suter W, Cox RH (1994). Carcinogenicity and mutagenicity studies with fluvastatin, a new, entirely synthetic HMG-CoA reductase inhibitor. Fundam Appl Toxicol.

[103] Prado CM, Antoun S, Sawyer MB, Baracos VE (2011). Two faces of drug therapy in cancer: drug-related lean tissue loss and its adverse consequences to survival and toxicity. Curr Opin Clin Nutr Metab Care.

[104] Song S, Xie M, Scott AW, Jin J, Ma L, Dong X, Skinner HD, Johnson RL, Ding S, Ajani JA (2018). A novel YAP1 inhibitor targets CSC-enriched radiation-resistant cells and exerts strong antitumor activity in esophageal adenocarcinoma. Mol Cancer Ther.

[105] Solmi M, Murru A, Pacchiarotti I, Undurraga J, Veronese N, Fornaro M, Stubbs B, Monaco F, Vieta E, Seeman MV (2017). Safety, tolerability, and risks associated with first- and second-generation antipsychotics: a state-of-the-art clinical review. Ther Clin Risk Manag.

[106] Zheng L, Xiang C, Li X, Guo Q, Gao L, Ni H, Xia Y, Xi T (2018). STARD13-correlated ceRNA network-directed inhibition on YAP/TAZ activity suppresses stemness of breast cancer via co-regulating Hippo and Rho-GTPase/F-actin signaling. J Hematol Oncol.

[107] Park JH, Shin JE, Park HW (2018). The role of Hippo pathway in cancer stem cell biology. Mol Cells.

[108] Giraud J, Molina-Castro S, Seeneevassen L, Sifré E, Izotte J, Tiffon C, Staedel C, Boeuf H, Fernandez S, Barthelemy P (2019). Verteporfin targeting YAP1/TAZ-TEAD transcriptional activity inhibits the tumorigenic properties of gastric cancer stem cells. Int J Cancer.

[109] Song S, Ajani JA, Honjo S, Maru DM, Chen Q, Scott AW, Heallen TR, Xiao L, Hofstetter WL, Weston B (2014). Hippo coactivator YAP1 upregulates SOX9 and endows esophageal cancer cells with stem-like properties. Cancer Res.

[110] Zhao S, Xu K, Jiang R, Li DY, Guo XX, Zhou P, Tang JF, Li LS, Zeng D, Hu L, et al. Evodiamine inhibits proliferation and promotes apoptosis of hepatocellular carcinoma cells via the Hippo-yes-associated protein signaling pathway. Life Sci. 2020;117424. 10.1016/j.lfs.2020.117424 32057900

[111] Kim H, Yu Y, Choi S, Lee H, Yu J, Lee JH, Kim WY (2019). Evodiamine eliminates colon cancer stem cells via suppressing Notch and Wnt signaling. Molecules.

[112] Qin J, Shi H, Xu Y, Zhao F, Wang Q (2018). Tanshinone IIA inhibits cervix carcinoma stem cells migration and invasion via inhibiting YAP transcriptional activity. Biomed Pharmacother.

[113] Zhao W, Wu M, Cui L, Du W (2019). Limonin attenuates the stemness of cervical carcinoma cells by promoting YAP nuclear-cytoplasmic translocation. Food Chem Toxicol.

[114] Tanaka K, Osada H, Murakami-Tonami Y, Horio Y, Hida T, Sekido Y (2017). Statin suppresses Hippo pathway-inactivated malignant mesothelioma cells and blocks the YAP/CD44 growth stimulatory axis. Cancer Lett.

[115] Koohestanimobarhan Soheila, Salami Siamak, Imeni Vahideh, Mohammadi Zeinab, Bayat Omid (2018). Lipophilic statins antagonistically alter the major epithelial‐to‐mesenchymal transition signaling pathways in breast cancer stem–like cells via inhibition of the mevalonate pathway. Journal of Cellular Biochemistry.

[116] Yang CE, Lee WY, Cheng HW, Chung CH, Mi FL, Lin CW (2019). The antipsychotic chlorpromazine suppresses YAP signaling, stemness properties, and drug resistance in breast cancer cells. Chem Biol Interact.

[117] Al-Bari MA (2015). Chloroquine analogues in drug discovery: new directions of uses, mechanisms of actions and toxic manifestations from malaria to multifarious diseases. J Antimicrob Chemother.

[118] Shi TT, Yu XX, Yan LJ, Xiao HT (2017). Research progress of hydroxychloroquine and autophagy inhibitors on cancer. Cancer Chemother Pharmacol.

[119] Brana I, Ocana A, Chen EX, Razak AR, Haines C, Lee C, Douglas S, Wang L, Siu LL, Tannock IF (2014). A phase I trial of pantoprazole in combination with doxorubicin in patients with advanced solid tumors: evaluation of pharmacokinetics of both drugs and tissue penetration of doxorubicin. Invest New Drugs.

[120] Dai S, Wang B, Li W, Wang L, Song X, Guo C, Li Y, Liu F, Zhu F, Wang Q (2016). Systemic application of 3-methyladenine markedly inhibited atherosclerotic lesion in ApoE(-/-) mice by modulating autophagy, foam cell formation and immune-negative molecules. Cell Death Dis.

[121] Huang M, Tang SN, Upadhyay G, Marsh JL, Jackman CP, Srivastava RK, Shankar S (2014). Rottlerin suppresses growth of human pancreatic tumors in nude mice, and pancreatic cancer cells isolated from Kras(G12D) mice. Cancer Lett.

[122] Nazio F, Bordi M, Cianfanelli V, Locatelli F, Cecconi F (2019). Autophagy and cancer stem cells: molecular mechanisms and therapeutic applications. Cell Death Differ.

[123] Auberger P, Puissant A (2017). Autophagy, a key mechanism of oncogenesis and resistance in leukemia. Blood.

[124] Liang DH, Choi DS, Ensor JE, Kaipparettu BA, Bass BL, Chang JC (2016). The autophagy inhibitor chloroquine targets cancer stem cells in triple negative breast cancer by inducing mitochondrial damage and impairing DNA break repair. Cancer Lett.

[125] Hao C, Liu G, Tian G (2019). Autophagy inhibition of cancer stem cells promotes the efficacy of cisplatin against non-small cell lung carcinoma. Ther Adv Respir Dis.

[126] Rothe K, Porter V, Jiang X (2019). Current outlook on autophagy in human leukemia: foe in cancer stem cells and drug resistance, friend in new therapeutic interventions. Int J Mol Sci.

[127] Horne GA, Stobo J, Kelly C, Mukhopadhyay A, Latif AL, Dixon-Hughes J, McMahon L, Cony-Makhoul P, Byrne J, Smith G, et al. A randomised phase II trial of hydroxychloroquine and imatinib versus imatinib alone for patients with chronic myeloid leukaemia in major cytogenetic response with residual disease. Leukemia. 2020. 10.1038/s41375-019-0700-9 PMC7224085 31925317

[128] Hansen AR, Tannock IF, Templeton A, Chen E, Evans A, Knox J, Prawira A, Sridhar SS, Tan S, Vera-Badillo F (2019). Pantoprazole affecting docetaxel resistance pathways via autophagy (PANDORA): phase II trial of high dose pantoprazole (autophagy inhibitor) with docetaxel in metastatic castration-resistant prostate cancer (mCRPC). Oncologist.

[129] Feng S, Zheng Z, Feng L, Yang L, Chen Z, Lin Y, Gao Y, Chen Y (2016). Proton pump inhibitor pantoprazole inhibits the proliferation, selfrenewal and chemoresistance of gastric cancer stem cells via the EMT/betacatenin pathways. Oncol Rep.

[130] Yang H, Zheng Y, Zhang Y, Cao Z, Jiang Y (2017). Mesenchymal stem cells derived from multiple myeloma patients protect against chemotherapy through autophagy-dependent activation of NF-kappaB signaling. Leuk Res.

[131] Kumar D, Shankar S, Srivastava RK (2013). Rottlerin-induced autophagy leads to the apoptosis in breast cancer stem cells: molecular mechanisms. Mol Cancer.

[132] Torti SV, Torti FM (2013). Iron and cancer: more ore to be mined. Nat Rev Cancer.

[133] El Hout M, Dos Santos L, Hamai A, Mehrpour M (2018). A promising new approach to cancer therapy: targeting iron metabolism in cancer stem cells. Semin Cancer Biol.

[134] Shah R, Shchepinov MS, Pratt DA (2018). Resolving the role of lipoxygenases in the initiation and execution of ferroptosis. ACS central science.

[135] Conrad M, Kagan VE, Bayir H, Pagnussat GC, Head B, Traber MG, Stockwell BR (2018). Regulation of lipid peroxidation and ferroptosis in diverse species. Genes Dev.

[136] Stoyanovsky DA, Tyurina YY, Shrivastava I, Bahar I, Tyurin VA, Protchenko O, Jadhav S, Bolevich SB, Kozlov AV, Vladimirov YA (2019). Iron catalysis of lipid peroxidation in ferroptosis: regulated enzymatic or random free radical reaction?. Free Radic Biol Med.

[137] Recalcati S, Gammella E, Cairo G (2019). Dysregulation of iron metabolism in cancer stem cells. Free Radic Biol Med.

[138] Chanvorachote P, Luanpitpong S (2016). Iron induces cancer stem cells and aggressive phenotypes in human lung cancer cells. Am J Physiol Cell Physiol.

[139] Raggi C, Gammella E, Correnti M, Buratti P, Forti E, Andersen JB, Alpini G, Glaser S, Alvaro D, Invernizzi P (2017). Dysregulation of iron metabolism in cholangiocarcinoma stem-like cells. Sci Rep.

[140] Schonberg DL, Miller TE, Wu Q, Flavahan WA, Das NK, Hale JS, Hubert CG, Mack SC, Jarrar AM (2015). Karl RT et al: preferential iron trafficking characterizes glioblastoma stem-like cells. Cancer Cell.

[141] Ishimoto T, Nagano O, Yae T, Tamada M, Motohara T, Oshima H, Oshima M, Ikeda T, Asaba R, Yagi H (2011). CD44 variant regulates redox status in cancer cells by stabilizing the xCT subunit of system xc(-) and thereby promotes tumor growth. Cancer Cell.

[142] Liang C, Zhang X, Yang M, Dong X (2019). Recent progress in ferroptosis inducers for cancer therapy. Adv Mater.

[143] Mai TT, Hamai A, Hienzsch A, Caneque T, Muller S, Wicinski J, Cabaud O, Leroy C, David A, Acevedo V (2017). Salinomycin kills cancer stem cells by sequestering iron in lysosomes. Nat Chem.

[144] Li L, Cui D, Ye L, Li Y, Zhu L, Yang L, Bai B, Nie Z, Gao J, Cao Y (2017). Codelivery of salinomycin and docetaxel using poly(D,L-lactic-co-glycolic acid)-poly(ethylene glycol) nanoparticles to target both gastric cancer cells and cancer stem cells. Anticancer Drugs.

[145] Antoszczak M (2019). A medicinal chemistry perspective on salinomycin as a potent anticancer and anti-CSCs agent. Eur J Med Chem.

[146] Zhao Y, Zhao W, Lim YC, Liu T (2019). Salinomycin-loaded gold nanoparticles for treating cancer stem cells by ferroptosis-induced cell death. Mol Pharm.

[147] Versini A, Colombeau L, Hienzsch A, Gaillet C, Retailleau P, Debieu S, Muller S, Caneque T, Rodriguez R: Salinomycin derivatives kill breast cancer stem cells via lysosomal iron targeting. Chemistry 2020. 10.1002/chem.202000335. 10.1002/chem.202000335 32083773

[148] Turcu AL, Versini A, Khene N, Gaillet C, Caneque T, Muller S, Rodriguez R. DMT1 inhibitors kill cancer stem cells by blocking lysosomal iron translocation. Chemistry 2020. 10.1002/chem.202000159. 10.1002/chem.202000159 32083771

[149] Basuli D, Tesfay L, Deng Z, Paul B, Yamamoto Y, Ning G, Xian W, McKeon F, Lynch M, Crum CP (2017). Iron addiction: a novel therapeutic target in ovarian cancer. Oncogene.

[150] Buccarelli M, Marconi M, Pacioni S, De Pascalis I, D'Alessandris QG, Martini M, Ascione B, Malorni W, Larocca LM, Pallini R (2018). Inhibition of autophagy increases susceptibility of glioblastoma stem cells to temozolomide by igniting ferroptosis. Cell Death Dis.

[151] Chen GQ, Benthani FA, Wu J, Liang D, Bian ZX, Jiang X (2020). Artemisinin compounds sensitize cancer cells to ferroptosis by regulating iron homeostasis. Cell Death Differ.

[152] Cao L, Duanmu W, Yin Y, Zhou Z, Ge H, Chen T, Tan L, Yu A, Hu R, Fei L (2014). Dihydroartemisinin exhibits anti-glioma stem cell activity through inhibiting p-AKT and activating caspase-3. Pharmazie.

[153] Subedi A, Futamura Y, Nishi M, Ryo A, Watanabe N, Osada H (2016). High-throughput screening identifies artesunate as selective inhibitor of cancer stemness: involvement of mitochondrial metabolism. Biochem Biophys Res Commun.

[154] Eling N, Reuter L, Hazin J, Hamacher-Brady A, Brady NR (2015). Identification of artesunate as a specific activator of ferroptosis in pancreatic cancer cells. Oncoscience.

[155] Su Yanwei, Zhao Bin, Zhou Liangfu, Zhang Zheyuan, Shen Ying, Lv Huanhuan, AlQudsy Luban Hamdy Hameed, Shang Peng (2020). Ferroptosis, a novel pharmacological mechanism of anti-cancer drugs. Cancer Letters.

[156] Sadhukha T, Niu L, Wiedmann TS, Panyam J (2013). Effective elimination of cancer stem cells by magnetic hyperthermia. Mol Pharm.

[157] Bullivant JP, Zhao S, Willenberg BJ, Kozissnik B, Batich CD, Dobson J (2013). Materials characterization of feraheme/ferumoxytol and preliminary evaluation of its potential for magnetic fluid hyperthermia. Int J Mol Sci.

[158] Chen B, Xing J, Li M, Liu Y, Ji M (2020). DOX@ferumoxytol-medical chitosan as magnetic hydrogel therapeutic system for effective magnetic hyperthermia and chemotherapy in vitro. Colloids Surf B Biointerfaces.

[159] Antoszczak M (2019). A comprehensive review of salinomycin derivatives as potent anticancer and anti-CSCs agents. Eur J Med Chem.

[160] Zhao B, Li X, Wang Y, Shang P (2018). Iron-dependent cell death as executioner of cancer stem cells. J Exp Clin Cancer Res.

[161] Azad GK, Tomar RS (2014). Ebselen, a promising antioxidant drug: mechanisms of action and targets of biological pathways. Mol Biol Rep.

[162] Zhang Z, Kodumuru V, Sviridov S, Liu S, Chafeev M, Chowdhury S, Chakka N, Sun J, Gauthier SJ, Mattice M (2012). Discovery of benzylisothioureas as potent divalent metal transporter 1 (DMT1) inhibitors. Bioorg Med Chem Lett.

[163] Stepanovic A, Nikitovic M (2018). Severe hematologic temozolomide-related toxicity and lifethreatening infections. J BUON.

[164] Luo Y, Che MJ, Liu C, Liu HG, Fu XW, Hou YP (2018). Toxicity and related mechanisms of dihydroartemisinin on porcine oocyte maturation in vitro. Toxicol Appl Pharmacol.

[165] Li H, Xu K, Pian G, Sun S (2019). Artesunate and sorafenib: combinatorial inhibition of liver cancer cell growth. Oncol Lett.

[166] Slezakova S, Ruda J: Anticancer activity of artemisinin and its derivatives. Anticancer Res 2017, 37(11). 10.21873/anticanres.12046 29061778

[167] Kaittanis C, Shaffer TM, Ogirala A, Santra S, Perez JM, Chiosis G, Li Y, Josephson L, Grimm J (2014). Environment-responsive nanophores for therapy and treatment monitoring via molecular MRI quenching. Nat Commun.

[168] Nguyen KL, Yoshida T, Kathuria-Prakash N, Zaki IH, Varallyay CG, Semple SI, Saouaf R, Rigsby CK, Stoumpos S, Whitehead KK (2019). Multicenter safety and practice for off-label diagnostic use of ferumoxytol in MRI. Radiology.

[169] Shepshelovich D, Rozen-Zvi B, Avni T, Gafter U, Gafter-Gvili A (2016). Intravenous versus oral iron supplementation for the treatment of anemia in CKD: an updated systematic review and meta-analysis. American journal of kidney diseases : the official journal of the National Kidney Foundation.

[170] Miller RC, Petereit DG, Sloan JA, Liu H, Martenson JA, Bearden JD, Sapiente R, Seeger GR, Mowat RB, Liem B (2016). N08C9 (Alliance): a phase 3 randomized study of sulfasalazine versus placebo in the prevention of acute diarrhea in patients receiving pelvic radiation therapy. Int J Radiat Oncol Biol Phys.

[171] Hangauer MJ, Viswanathan VS, Ryan MJ, Bole D, Eaton JK, Matov A, Galeas J, Dhruv HD, Berens ME, Schreiber SL (2017). Drug-tolerant persister cancer cells are vulnerable to GPX4 inhibition. Nature.

[172] Viswanathan VS, Ryan MJ, Dhruv HD, Gill S, Eichhoff OM, Seashore-Ludlow B, Kaffenberger SD, Eaton JK, Shimada K, Aguirre AJ (2017). Dependency of a therapy-resistant state of cancer cells on a lipid peroxidase pathway. Nature.

[173] Jung Y, Park H, Zhao HY, Jeon R, Ryu JH, Kim WY (2014). Systemic approaches identify a garlic-derived chemical, Z-ajoene, as a glioblastoma multiforme cancer stem cell-specific targeting agent. Mol Cells.

[174] Kim TY, Han HS, Lee KW, Zang DY, Rha SY, Park YI, Kim JS, Lee KH, Park SH, Song EK (2019). A phase I/II study of poziotinib combined with paclitaxel and trastuzumab in patients with HER2-positive advanced gastric cancer. Gastric Cancer.

[175] Zhu Y, Warin RF, Soroka DN, Chen H, Sang S (2013). Metabolites of ginger component [6]-shogaol remain bioactive in cancer cells and have low toxicity in normal cells: chemical synthesis and biological evaluation. PLoS One.

[176] Lee H, Kim JW, Choi DK, Yu JH, Kim JH, Lee DS, Min SH (2020). Poziotinib suppresses ovarian cancer stem cell growth via inhibition of HER4-mediated STAT5 pathway. Biochem Biophys Res Commun.

[177] Ray A, Vasudevan S, Sengupta S (2015). 6-Shogaol inhibits breast cancer cells and stem cell-like spheroids by modulation of Notch signaling pathway and induction of autophagic cell death. PLoS One.

[178] Dreesen O, Brivanlou AH (2007). Signaling pathways in cancer and embryonic stem cells. Stem Cell Rev.

